# Artificial intelligence for precision therapeutics in age-related macular degeneration: current advances, challenges, and future directions

**DOI:** 10.3389/frai.2026.1822604

**Published:** 2026-08-17

**Authors:** Mini Han Wang, Simon Ming Yuen Lee, Yapeng Wang, José C. Alves, Ruitao Xie, Yaqing He, Guanghui Hou, Xiaoxiao Fang, Yang Yu, Xiaodong Cai, Shuai Zheng, Jin Liu, Chonin Cheang, Kai Ian Kuok, Shuai Qin

**Affiliations:** 1 Shenzhen University of Advanced Technology, Shenzhen, China; 2 Zhuhai People's Hospital (The Affiliated Hospital of Beijing Institute of Technology, Zhuhai Clinical Medical College of Jinan University), Zhuhai, Guangdong, China; 3 Faculty of Medicine, The Chinese University of Hong Kong, Hong Kong, Hong Kong SAR, China; 4 Zhuhai Institute of Advanced Technology, Chinese Academy of Sciences, Zhuhai, Guangdong, China; 5 Department of Food Science and Nutrition, The Hong Kong Polytechnic University, Hong Kong, Hong Kong SAR, China; 6 Faculty of Applied Sciences, Macao Polytechnic University, Macao, Macao SAR, China; 7 Faculty of Business, City University of Macau, Macao, Macao SAR, China; 8 Smart City Division, Hong Kong Productivity Council, Hong Kong, Hong Kong SAR, China; 9 Aier Eye Hospital of Zhuhai, Zhuhai, Guangdong, China; 10 Department of Orthopedic Spinal Surgery, Nanfang Hospital, Southern Medical University, Guangzhou, China; 11 Hunan Provincial Key Lab on Bioinformatics, School of Computer Science and Engineering, Central South University, Changsha, China; 12 Macau Yinkui Hospital, Macao, Macao SAR, China; 13 Macau Society for Health Economics, Macao, Macao SAR, China; 14 Xiaoao Technology Co., Ltd., Macao, Macao SAR, China; 15 Department of Ophthalmology, The Third Affiliated Hospital of Southern Medical University, Guangzhou, China

**Keywords:** age-related macular degeneration, artificial intelligence, explainable artificial intelligence, multimodal imaging, precision therapeutics

## Abstract

Age-related macular degeneration (AMD) is a leading cause of irreversible vision loss worldwide and is characterized by substantial clinical, imaging, and molecular heterogeneity that complicates disease prediction and therapeutic management. Recent advances in artificial intelligence (AI) and precision therapeutics have created new opportunities for more individualized and data-driven AMD care. AI models trained on multimodal datasets—including fundus photography, optical coherence tomography (OCT), optical coherence tomography angiography (OCTA), genetic susceptibility loci (e.g., CFH, ARMS2/HTRA1, C3, CFI, and APOE), and longitudinal clinical information—have demonstrated promising capability in early disease detection, progression forecasting, biomarker identification, and prediction of treatment response. These developments align closely with emerging precision therapeutic strategies, including optimized anti-vascular endothelial growth factor (anti-VEGF) regimens, complement-targeted therapies, gene-based interventions, and stem cell-associated regenerative approaches. This review provides a translational overview of AI-enabled precision therapeutics in AMD, with emphasis on multimodal biomarker integration, individualized therapeutic stratification, longitudinal disease monitoring, and clinically interpretable AI systems. Importantly, we further propose a Five-Level Clinical Readiness and Translational Utility Framework for AI in AMD Precision Therapeutics, categorizing AI applications according to evidence strength, clinical maturity, validation status, interpretability, and real-world implementation potential. The framework distinguishes near-reference-standard imaging AI systems, advanced clinical decision-support tools, emerging multimodal precision therapeutic AI, supportive workflow-oriented AI systems, and currently limited or unsuitable AI applications. Despite substantial progress, important translational barriers remain, including limited external validation, retrospective study designs, dataset heterogeneity, domain shift, insufficient explainability, regulatory uncertainty, and challenges related to workflow integration and real-world clinical deployment. Future advances in multimodal longitudinal AI, explainable AI, federated learning, digital health platforms, and multi-omics integration may facilitate a transition from reactive disease management toward more proactive, predictive, and personalized ophthalmic care. Collectively, AI-enabled precision therapeutics may help establish a more scalable and clinically integrated framework for individualized AMD management and future precision ophthalmology.

## 1 Introduction

### 1.1 Age-related macular degeneration overview

Age-related macular degeneration (AMD) is a leading cause of irreversible vision loss among older adults worldwide, currently affecting nearly 200 million individuals, with global prevalence projected to approach 288 million by 2040 (Shirian et al., 2025). The disease represents a major public health challenge because of its progressive course, substantial biological heterogeneity (Hushmandi et al., 2025), and highly variable therapeutic response patterns (Reeve et al., 2025). Despite major advances in ophthalmic imaging and intravitreal therapeutics, effective long-term disease control remains challenging in a significant proportion of patients. These limitations have accelerated growing interest in precision medicine and artificial intelligence (AI)-enabled approaches capable of improving individualized diagnosis (Frank-Publig et al., 2025), prognostication, and therapeutic optimization.

Clinically, AMD encompasses multiple disease stages and phenotypes, including early/intermediate dry AMD, neovascular AMD (nAMD), and advanced geographic atrophy (GA). Each stage presents distinct clinical decision-making challenges, including early disease screening, progression prediction, conversion risk assessment from dry AMD to nAMD, optimization of anti-vascular endothelial growth factor (anti-VEGF) therapy, and longitudinal monitoring of atrophic progression. Increasingly, AI systems are being developed to address these specific clinical decision points and facilitate more personalized therapeutic strategies across the AMD disease spectrum (Parmar et al., 2025).

Pathologically, AMD is characterized by progressive degeneration of the central retina, particularly involving the retinal pigment epithelium (RPE), photoreceptors, Bruch's membrane, and choriocapillaris. The non-exudative (dry or atrophic) form, which accounts for approximately 80–90% of cases, is characterized by gradual accumulation of drusen beneath the RPE together with progressive retinal atrophy and photoreceptor degeneration (Zhang et al., 2025). Although dry AMD generally progresses more slowly, advanced disease may result in GA and irreversible central visual loss. By contrast, the exudative (wet or neovascular) form is characterized by pathological choroidal neovascularization (CNV), leading to vascular leakage, hemorrhage, fibrovascular scarring, and rapid visual deterioration if untreated.

Diagnosis and monitoring of AMD rely heavily on multimodal retinal imaging. Fundus photography enables documentation of drusen and pigmentary abnormalities, while optical coherence tomography (OCT) provides high-resolution cross-sectional visualization of retinal microstructure, facilitating detection of intraretinal fluid, subretinal fluid, pigment epithelial detachment, and early atrophic alterations. Optical coherence tomography angiography (OCTA) further enhances non-invasive visualization of choroidal neovascular networks, complementing fluorescein angiography and indocyanine green angiography for assessment of vascular activity and lesion characterization. Nevertheless, diagnostic interpretation remains limited by interobserver variability, imaging artifacts, inconsistent grading standards, and the inability of conventional imaging alone to fully capture the molecular and phenotypic complexity underlying disease progression.

Therapeutic strategies for AMD are highly dependent on disease subtype and progression stage. In nAMD, intravitreal anti-VEGF agents—including ranibizumab, aflibercept, and brolucizumab—have substantially improved visual outcomes and remain the cornerstone of treatment (Shirian et al., 2025). However, therapeutic responses remain heterogeneous, with many patients requiring frequent injections and some demonstrating incomplete or suboptimal response. In parallel, recent advances in complement-targeted therapy have significantly expanded treatment options for GA secondary to AMD. Intravitreal complement inhibitors, including pegcetacoplan (targeting C3) and avacincaptad pegol (targeting C5), have received FDA approval after demonstrating the ability to slow GA lesion progression in phase III clinical trials. Despite these advances, currently available therapies remain limited by incomplete functional restoration, treatment burden, and variable efficacy, underscoring the continuing need for more individualized and biologically informed therapeutic strategies.

Emerging therapeutic development is increasingly extending beyond conventional VEGF suppression toward broader precision therapeutic paradigms. Investigational approaches include gene therapies designed to provide sustained intraocular anti-VEGF expression, broader angiogenic pathway modulation, sustained-release delivery systems, regenerative stem cell-associated interventions, and therapies targeting oxidative stress, inflammation, and regulated cell death pathways. In addition, novel biomaterial-based delivery systems, including biomimetic nanoparticle platforms and microneedle-assisted retinal drug delivery strategies, are being explored to improve therapeutic durability, reduce treatment burden, and enhance retinal targeting efficiency (Liu J. et al., 2025).

Growing evidence further highlights the multifactorial biological mechanisms underlying AMD progression, including oxidative stress, chronic inflammation, mitochondrial dysfunction, complement dysregulation, and regulated cell death pathways such as apoptosis, ferroptosis, pyroptosis, autophagy, and necroptosis (Parmar et al., 2025). These findings have stimulated increasing interest in mechanism-oriented therapeutic strategies, including complement inhibition, antioxidant modulation, autophagy regulation, neuroprotection, and drug repurposing approaches. Landmark clinical trials such as AREDS and AREDS2 also demonstrated that antioxidant supplementation—including vitamins C and E, lutein, zeaxanthin, and related compounds—may reduce progression risk in selected patient populations, although therapeutic response variability remains substantial (Zhang et al., 2025).

Collectively, the biological heterogeneity, variable treatment response, and complex multimodal disease characteristics of AMD highlight the limitations of conventional “one-size-fits-all” management approaches. These challenges have increasingly positioned AMD as a major target for AI-enabled precision therapeutics integrating multimodal imaging, genomic susceptibility profiles, molecular biomarkers, and longitudinal clinical data to support more predictive, personalized, and proactive ophthalmic care.

### 1.2 Genetic and molecular susceptibility in AMD

Over the past two decades, advances in molecular genetics and genome-wide association studies (GWAS) have substantially improved understanding of the genetic architecture underlying age-related macular degeneration (AMD). Numerous susceptibility loci—including CFH, ARMS2/HTRA1, C3, CFI, and APOE—have been consistently associated with disease predisposition, progression, and variability in therapeutic response, highlighting the polygenic and multifactorial nature of AMD (Hushmandi et al., 2025; Reeve et al., 2025). These discoveries have strengthened interest in precision therapeutics aimed at tailoring interventions according to individual genetic, molecular, and phenotypic characteristics. Nevertheless, clinical translation remains limited because of the complexity of integrating multi-omic data into routine ophthalmic decision-making workflows.

Among the most extensively studied loci, CFH plays a central role in regulating the complement cascade and maintaining immune homeostasis within the retina. Variants affecting CFH function have been strongly associated with aberrant complement activation, chronic retinal inflammation, and increased AMD susceptibility. Recent large-scale genetic analyses involving more than 12,000 AMD cases and over 460,000 controls further refined the CFH locus and identified multiple protective haplotypes associated with CFHR5 variants, suggesting that modulation of complement regulatory proteins may influence retinal structural preservation and disease risk (Reeve et al., 2025). These findings reinforce the biological importance of complement dysregulation in AMD pathogenesis and provide mechanistic support for emerging complement-targeted therapeutic strategies.

In parallel, susceptibility variants within the ARMS2/HTRA1 locus on chromosome 10q26 have been strongly associated with oxidative stress, extracellular matrix remodeling, angiogenesis, and neovascular AMD development (Katschke et al., 2025). Additional transcriptomic and long non-coding RNA analyses further suggest that dysregulated transcriptional control within the ARMS2-HTRA1 region may contribute to disease susceptibility through altered inflammatory and angiogenic signaling pathways (Zhang et al., 2025). Likewise, complement-related genes such as C3 and CFI further support the central role of innate immune dysregulation in AMD progression, while APOE polymorphisms implicate lipid transport and metabolic dysfunction in drusen formation and early retinal degeneration. Collectively, these findings emphasize that AMD arises through complex interactions among inflammatory, angiogenic, oxidative, and metabolic pathways rather than through a single pathogenic mechanism.

The molecular heterogeneity of AMD has important translational implications for precision therapeutics. Dysregulation of the complement system contributes to persistent inflammation at the retinal pigment epithelium (RPE)-Bruch's membrane-choroid interface, while altered lipid metabolism promotes drusen accumulation and progressive RPE dysfunction. Concurrently, pro-angiogenic signaling pathways, particularly those mediated by vascular endothelial growth factor (VEGF), drive the development of choroidal neovascularization (CNV) in neovascular AMD. These interconnected mechanisms have stimulated growing interest in biologically targeted therapeutic strategies, including complement inhibitors, anti-inflammatory approaches, antioxidant modulation, and individualized anti-VEGF treatment optimization guided by molecular profiling.

Importantly, genetic susceptibility alone does not fully explain AMD onset or progression. Environmental and lifestyle factors—including smoking, diet, cardiovascular disease, and metabolic comorbidities—interact dynamically with genetic background to modulate disease risk (He et al., 2025). Emerging mechanistic evidence suggests that smoking may exacerbate AMD progression through inflammatory and angiogenic pathways involving Sema4D-PlexinB1 signaling, immune cell migration, and disruption of vascular homeostasis, thereby potentially reducing responsiveness to anti-VEGF therapy (He et al., 2025). These observations further highlight the importance of integrating environmental, molecular, and clinical information into future risk prediction and therapeutic frameworks.

Despite major advances in AMD genetics, several translational barriers remain. Many susceptibility loci identified through GWAS exhibit relatively modest individual effect sizes and lack comprehensive mechanistic validation, limiting their immediate clinical applicability for patient stratification. In addition, substantial heterogeneity exists across populations, disease phenotypes, and environmental exposures, complicating the development of universally applicable predictive models. Consequently, most genetic biomarkers have not yet been incorporated into routine diagnostic or therapeutic decision-making pathways.

Future progress will likely require integrative multimodal frameworks capable of synthesizing genomic, molecular, imaging, and longitudinal clinical data into clinically actionable predictive systems. In this context, artificial intelligence (AI) and multimodal machine learning approaches may provide powerful tools for integrating complex biological information, improving individualized risk prediction, identifying novel therapeutic targets, and facilitating the development of more precise and personalized AMD management strategies.

### 1.3 Artificial intelligence and precision therapeutics in AMD: toward translational precision ophthalmology

Artificial intelligence has emerged as a transformative approach for addressing the substantial biological, diagnostic, and therapeutic complexity of age-related macular degeneration. AMD is characterized by considerable heterogeneity in genetic susceptibility, molecular pathways, retinal phenotypes, disease trajectories, and therapeutic responses. By enabling automated analysis and integration of high-dimensional multimodal data—including retinal imaging, genomic information, molecular biomarkers, electronic health records, and longitudinal clinical observations—AI systems provide powerful tools for identifying clinically meaningful patterns that may support individualized disease characterization, risk prediction, and therapeutic optimization.

Recent advances in deep learning, multimodal machine learning, and explainable artificial intelligence (XAI) have expanded the role of AI beyond conventional diagnostic classification toward clinically actionable decision-support applications. In AMD, emerging AI approaches have demonstrated potential for biomarker discovery, progression forecasting, conversion risk prediction from dry AMD to neovascular AMD, anti-vascular endothelial growth factor (anti-VEGF) prediction of treatment response, recurrence risk estimation, geographic atrophy progression assessment, and individualized monitoring strategies. Importantly, these applications do not represent precision therapeutics by themselves; rather, they serve as enabling technologies that generate patient-specific information to support therapeutic stratification, treatment selection, and adaptive disease management.

In this review, precision therapeutics in AMD is defined as an individualized therapeutic paradigm that integrates patient-specific biological, imaging, genetic, molecular, and longitudinal clinical information to optimize therapeutic decision-making. Importantly, precision therapeutics should not be interpreted solely as the development of novel gene-based interventions or regenerative therapies. Although emerging approaches such as gene therapy, stem cell-associated regeneration, and molecularly targeted treatments represent important components of future precision medicine, current precision therapeutic strategies also encompass the individualized application of established therapies. Examples include AI-assisted prediction of anti-VEGF treatment response, identification of likely responders and non-responders, optimization of treatment intervals, prediction of recurrence risk, and biomarker-guided therapeutic adaptation. Therefore, precision therapeutics in AMD represents a continuum ranging from optimization of existing clinical interventions to the development of next-generation biologically targeted therapies.

Retinal imaging plays a central role within this precision therapeutic paradigm. Although imaging modalities such as fundus photography, optical coherence tomography (OCT), and optical coherence tomography angiography (OCTA) are not therapeutic interventions themselves, they provide clinically accessible and quantitative biomarkers that capture disease activity, structural changes, vascular alterations, and treatment responses. Consequently, imaging-based AI systems represent an essential foundation for precision therapeutics by enabling objective phenotyping, longitudinal monitoring, and individualized prediction of therapeutic outcomes. Integration of imaging-derived biomarkers with genomic, molecular, and clinical information may further facilitate a transition from population-based treatment strategies toward more personalized and biologically informed AMD management.

Previous reviews, including our earlier publications, have extensively summarized AI applications in AMD diagnosis, retinal image analysis, and predictive modeling. In contrast, the present review specifically focuses on the translational convergence between AI-enabled multimodal analytics and precision therapeutics in AMD. Rather than evaluating AI solely according to diagnostic performance metrics, this review emphasizes how AI may facilitate individualized therapeutic decision-making through prediction of treatment response, therapeutic stratification, multimodal biomarker integration, explainable decision support, and clinician-centered longitudinal management. Furthermore, emerging therapeutic directions, including complement-targeted therapies, gene-based approaches, regenerative strategies, and AI-guided optimization of existing treatments, are discussed within the broader framework of precision ophthalmology.

Despite rapid advances, substantial challenges remain before AI-enabled precision therapeutics can be routinely implemented in clinical practice. Algorithmic performance alone does not guarantee clinical utility, as real-world deployment requires robust external validation, generalizability across diverse populations and imaging platforms, interpretability, regulatory compliance, workflow compatibility, and appropriate human oversight. Furthermore, current AI systems vary substantially in evidence strength, clinical maturity, and translational readiness. A clinically grounded approach is therefore required to distinguish mature AI technologies from emerging investigational systems and speculative applications.

**Figure 1:**
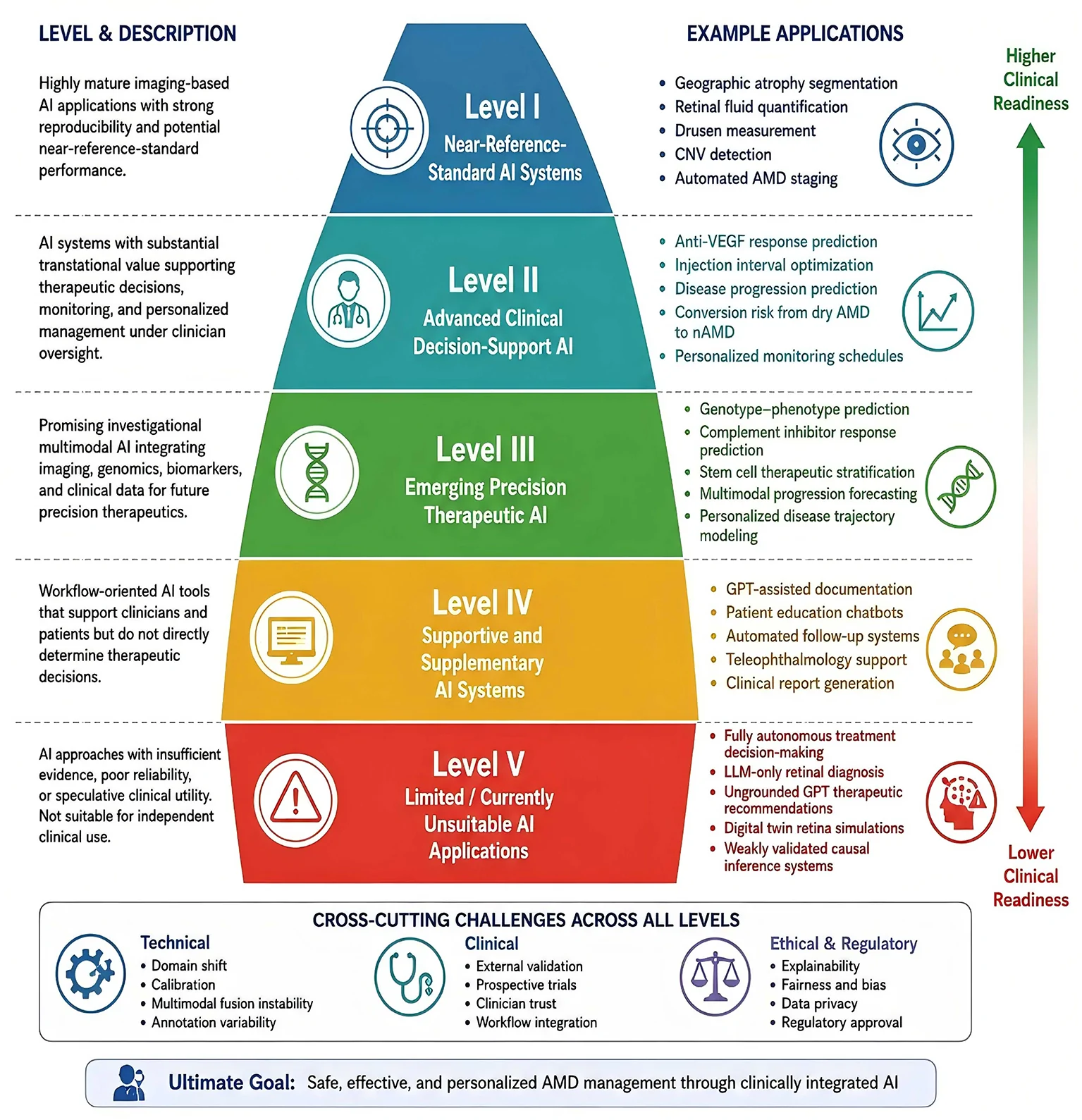
Five-level clinical readiness and translational utility framework for AI-enabled precision therapeutics in age-related macular degeneration.

To address this challenge, this study proposes a Five-Level Clinical Readiness and Translational Utility Framework for AI-enabled precision therapeutics in AMD (Figure 1 and Table 1). The framework was developed through a structured conceptual synthesis of representative AI studies involving AMD diagnosis, biomarker discovery, prediction of treatment response, prognosis, longitudinal monitoring, and multimodal precision medicine applications. Four major dimensions were considered during framework development: (1) clinical evidence strength, including validation strategy and study design; (2) technological maturity, including algorithmic robustness and multimodal integration capability; (3) clinical utility, including relevance to therapeutic decision-making and workflow integration; and (4) trustworthiness considerations, including explainability, reproducibility, uncertainty assessment, and regulatory readiness. These levels are not intended to represent a ranking of algorithmic performance alone, but rather reflect differences in clinical evidence, validation maturity, interpretability, therapeutic relevance, and feasibility for real-world implementation. Detailed discussion of each translational level is provided in Section 6.

**Table 1:** Summary of the five-level clinical readiness and translational utility framework for AI-enabled precision therapeutics in age-related macular degeneration.

Level	Classification	Evidence strength	Clinical readiness	Example applications
Level I	Near-reference-standard AI	Strong	High	GA segmentation, retinal fluid quantification
Level II	Advanced clinical decision-support AI	Moderate–strong	Moderate–high	Anti-VEGF response prediction, progression forecasting
Level III	Emerging precision therapeutic AI	Moderate	Emerging	Multimodal genomics-integrated prediction
Level IV	Supportive/supplementary AI	Limited–moderate	Supportive only	GPT-assisted documentation, teleophthalmology support
Level V	Limited or currently unsuitable AI	Weak/insufficient	Low	Fully autonomous treatment selection, speculative digital twins

Within this framework, AI applications are categorized into five translational levels: Level I, near-reference-standard imaging AI systems; Level II, advanced clinical decision-support AI systems; Level III, emerging precision therapeutic AI systems; Level IV, supportive and workflow-oriented AI systems; and Level V, limited or currently unsuitable AI applications. By distinguishing clinically actionable technologies from investigational or speculative approaches, this framework aims to provide a more balanced and clinically relevant interpretation of the evolving AI landscape in AMD precision therapeutics.

Finally, this review examines future directions toward clinically integrated precision ophthalmology, including multimodal longitudinal AI, federated learning, explainable AI, foundation models, human-AI collaboration, uncertainty-aware prediction, and individualized therapeutic adaptation strategies. Through the integration of AI with multimodal biological and clinical information, future AMD management may evolve from reactive treatment paradigms toward more predictive, proactive, and personalized approaches.

## 2 Literature search strategy and review methodology

A structured narrative literature review was conducted using four major electronic databases, including PubMed, Web of Science, Scopus, and Embase. Relevant articles published between January 2018 and May 2026 were systematically identified using combinations of keywords related to age-related macular degeneration (AMD), artificial intelligence (AI), precision therapeutics, and multimodal ophthalmic imaging. The primary search terms included “age-related macular degeneration,” “AMD,” “artificial intelligence,” “deep learning,” “machine learning,” “precision therapeutics,” “optical coherence tomography,” “OCT angiography,” “anti-VEGF response prediction,” “genetics,” and “multimodal imaging.”

Studies were considered eligible if they specifically focused on AMD and investigated AI-assisted diagnosis, disease prognosis, longitudinal monitoring, therapeutic response prediction, or precision treatment strategies. Studies exploring multimodal integration of ophthalmic imaging, genomic information, and clinical data were also included. Articles unrelated to AMD, conference abstracts lacking sufficient methodological detail, non-English publications, and studies without clear translational or clinical relevance were excluded from the review.

Following database retrieval, titles and abstracts were initially screened, followed by comprehensive full-text evaluation and narrative synthesis of eligible studies. Particular emphasis was placed on pivotal clinical trials, large multicenter investigations, externally validated AI systems, and authoritative systematic reviews or meta-analyses to ensure clinical relevance and scientific rigor.

The primary objective of this review was to synthesize current translational advances at the intersection of artificial intelligence and precision therapeutics in AMD, while critically discussing existing limitations, challenges in real-world clinical implementation, and future directions for personalized ophthalmic care.

## 3 Artificial intelligence as an enabling technology for AMD precision therapeutics

AI applications discussed in this section are not considered precision therapeutics by themselves. Rather, they represent enabling technologies that provide clinically actionable information for individualized therapeutic decision-making. In AMD, precision therapeutics requires the integration of AI-derived phenotypic, molecular, and longitudinal information to support patient stratification, treatment selection, response prediction, and adaptive disease management. Therefore, this section focuses on AI approaches that contribute to the development of precision therapeutic workflows rather than general diagnostic applications alone.

### 3.1 Artificial intelligence as an enabling technology for AMD precision therapeutics

AI has emerged as a transformative tool in ophthalmology (Michl et al., 2025), particularly in the early detection (Crincoli et al., 2024) and screening of AMD (Wang et al., 2023). By leveraging machine learning and deep learning algorithms, AI systems can analyze large-scale imaging datasets with accuracy and consistency, offering significant advantages over conventional human assessment (Savoy et al., 2024).

Fundus photography remains a widely used modality for AMD screening (Saha et al., 2019), and AI-based systems (Enzendorfer et al., 2025) have demonstrated high sensitivity and specificity in identifying drusen, pigmentary abnormalities, and other early disease features. More advanced imaging modalities, particularly OCT, provide cross-sectional visualization of retinal architecture, enabling AI models to detect subtle biomarkers (Li et al., 2025) of AMD, such as subretinal fluid, pigment epithelial detachment, and hyperreflective foci. In addition, the integration of OCTA has expanded the capability of AI algorithms to identify choroidal neovascularization non-invasively, improving the detection of early neovascular conversion. Automated interpretation of these multimodal images (Leng et al., 2023) not only enhances diagnostic accuracy but also reduces reliance on specialized graders, thereby increasing accessibility in both clinical and community-based settings.

Recent advances (Table 2) in artificial intelligence have generated a diverse portfolio of diagnostic strategies for AMD and related retinal disorders. These approaches span multiple imaging modalities and even systemic biomarkers, offering early detection, precise staging, and population-level screening opportunities.

**Table 2:** Summary of AI methods for early detection and screening, prediction of treatment response, and prognosis and monitoring of age-related macular degeneration.

References	AI model	Evidence source	Sample size	Clinical task	Validation Strategy	External validation	Key performance	Key limitations	Translational level
Saha et al. (2019)	CNNs (Inception-v3, ResNet50, InceptionResNet50)	SD-OCT B-scans	~20,000 scans; 153 patients	Early AMD biomarker detection (SDD, IHRF, hRF)	Internal holdout validation	No	Acc 86–89%	Single-center retrospective dataset; limited generalizability	Level I
Thakoor et al. (2022)	Multimodal 2D−3D CNNs	OCTA + OCT + B-scan flow images	501 eyes	AMD subtype classification and biomarker detection	Internal validation	No	Acc 94.7%	Limited external validation; device-specific imaging	Level I
Chen et al. (2023)	Ensemble CNN + ResNet50 + YOLOv3	OCT images	37,138 images; 775 patients	AMD/VMT classification and lesion localization	Patient-based holdout testing	No	AUC 98.1%; Acc ≥ 99%	Single-center study; lack of prospective deployment	Level I
Crincoli et al. (2024)	ResNet-101, DenseNet-201, soft-voting DL	OCT + OCTA	89 patients	Prediction of exudation risk in non-exudative MNV	Internal testing	No	Acc 94.4%	Small cohort; limited multicenter evidence	Level II
Savoy et al. (2024)	DL classifier for smartphone fundus imaging	AREDS + smartphone fundus photographs	>108,000 training images	Referable AMD detection	Internal testing + transfer learning	Partial	AUC 0.947–0.965	Population/device variability	Level I
Yeh et al. (2022)	HDF-Net multimodal CNN	OCT + clinical metadata	1,396 image series	Anti-VEGF response prediction	Internal validation	No	AUC 0.989	No multicenter prospective validation	Level II
Fu et al. (2021)	Automated Quantitative Optical Coherence Tomography (qOCT) biomarker extraction + ML	OCT biomarkers	926 eyes	Visual outcome prediction after anti-VEGF	Retrospective longitudinal analysis	No	*R*^2^ up to 0.79	Retrospective design; treatment heterogeneity	Level II
Han (2025)	DenseNet201	SD-OCT	517 patients	Anti-VEGF response and recurrence prediction	Internal validation	No	Acc 82%	Moderate recurrence prediction accuracy; single ethnicity cohort	Level II
Pawloff et al. (2023)	3D OCT DL segmentation	Multiplatform SD-OCT	41,906 scans	Automated IRF/SRF quantification	Clinical trial dataset validation	Yes	AUC up to 0.93	Residual prediction errors; workflow integration needed	Level I
Liu S. et al. (2025)	Improved LUNet + regression analysis	OCTA + clinical metadata	165 patients	Anti-VEGF response prediction	Retrospective single-center validation	No	Significant predictive biomarkers identified	Small sample size; limited external validation	Level II
Li et al. (2025)	ML metabolomic classifier	Serum metabolomics	547 participants	AMD diagnosis and staging	External validation phase included	Yes	AUC 0.962–1.000	Requires broader multicenter biomarker standardization	Level III
Das et al. (2019)	AMD-ResNet + LSTM	Fundus imaging + IoMT	>67,000 images	AMD staging and progression prediction	Internal testing	No	Acc 97.5%; AUROC 0.96–0.97	Proposed longitudinal prediction lacks prospective validation	Level II
Ejaz et al. (2024)	CNN architectures with transfer learning	Fundus photographs	~9,300 augmented images	Multi-retinal disease classification	Internal train/test split	No	Test Acc ~89%	Not AMD-specific; possible overfitting	Level IV

One line of research focuses on high-resolution OCT for early biomarker detection. Saha et al. (2019) applied deep convolutional neural networks—including Inception-v3, ResNet50, and InceptionResNet50—combined with ReLayNet pre-segmentation and transfer learning to ~20,000 spectral-domain OCT B-scans from 153 patients. Their models achieved 86–89% accuracy for identifying subretinal drusenoid deposits and hyperreflective foci, demonstrating that automated biomarker detection from OCT can feasibly support consistent, rapid staging and monitoring of early AMD in both clinical and community settings.

Expanding to multi-modal imaging, Thakoor et al. (2022) developed nine hybrid 2D−3D convolutional neural network architectures integrating OCTA, OCT structure, high-definition 5-line B-scan cubes, and 2D B-scan flow images. Their best multimodal models reached approximately 94.7% accuracy in distinguishing non-AMD, non-neovascular AMD, and neovascular AMD, while detecting key biomarkers such as choroidal neovascularization and geographic atrophy with high precision. This work shows how AI can emulate expert use of multiple imaging inputs, providing robust disease classification and biomarker detection for scalable early screening and disease staging.

Other scientific explorations have optimized decision-support pipelines for routine OCT workflows. Chen et al. (2023) reported a three-module deep learning system incorporating ensemble binary classification, disease classification, and YOLOv3-based lesion localization. Trained on more than 37,000 OCT images, this system achieved near-perfect accuracy for detecting AMD and vitreomacular traction while localizing lesions with recalls up to 98%. The multistage design outperformed single-step models and illustrates how AI can triage abnormal scans, enhance clinical throughput, and facilitate detailed interpretation.

A related direction aims at prognostic risk stratification. Crincoli et al. (2024) trained ResNet-101, Inception-ResNet-v2, and DenseNet-201 on OCT and OCTA images of eyes with non-exudative macular neovascularization, using ensemble major and soft voting. Their combined model predicted exudation risk within 2 years with 94.4% accuracy, surpassing human graders. Such tools can personalize follow-up intervals and enable timely intervention to prevent vision loss.

Beyond OCT, color fundus photography remains essential for population screening. Ejaz et al. (2024) applied three convolutional neural network architectures with extensive data augmentation to the RFMiD and RFMiD 2.0 datasets, successfully classifying diabetic retinopathy, media haze, optic disc cupping, and normal images with ~90% validation and ~89% test accuracy. In parallel, Savoy et al. (2024)) demonstrated that deep learning models trained on the Age-Related Eye Disease Study (AREDS) fundus database and fine-tuned on smartphone fundus images could detect referable AMD with an area under the receiver operating curve of about 0.95. These results underscore the feasibility of accurate, low-cost community screening using portable fundus cameras.

Finally, systemic biomarker approaches are emerging. Li et al. (2025) combined mass-spectrometry-based serum metabolomics with machine-learning classifiers to discover a three-metabolite panel—hypoxanthine, 2-furoylglycine, and 1-hexadecyl-2-azelaoyl-sn-glycero-3-phosphocholine—that achieved AUC values up to 1.0 for both AMD diagnosis and severity grading. The stability of these metabolites across repeated freeze-thaw cycles supports their potential as the basis for the first routine laboratory blood test for AMD, enabling non-invasive early detection and precision monitoring.

Collectively, these studies illustrate a rapidly evolving landscape in which deep learning and machine learning extend from OCT and fundus imaging to blood-based metabolomics. Together, they chart a path toward earlier diagnosis, individualized follow-up, and scalable screening of AMD and other blinding retinal diseases across diverse clinical and community environments.

Beyond detection, AI systems are increasingly being applied to predict the risk of disease progression, particularly the transition from early (Ejaz et al., 2024) or intermediate dry AMD to advanced stages, including geographic atrophy and neovascular AMD. Deep learning models (Kiruthika and Malathi, 2025) trained on longitudinal OCT and fundus datasets have shown the ability to identify imaging biomarkers predictive of progression, such as drusen volume changes, RPE irregularities, and early signs of neovascular activity (Chen et al., 2023). Some models incorporate multimodal data—including genetics, demographics, and lifestyle factors—into predictive frameworks, further enhancing their prognostic utility. By stratifying patients according to individualized risk, AI tools can support proactive monitoring, timely intervention, and more efficient allocation of clinical resources. These applications underscore the potential of AI to augment clinical workflows by facilitating early identification of AMD, forecasting disease trajectories, and ultimately supporting personalized management strategies.

### 3.2 AI for AMD prediction of treatment response

Although intravitreal anti-VEGF agents have revolutionized the management of neovascular AMD (Han, 2025), treatment outcomes remain heterogeneous. A proportion of patients exhibit substantial visual improvement, while others experience only partial stabilization or continued deterioration despite regular therapy. This variability underscores the need for predictive tools that can identify likely responders and non-responders before or early during treatment, thereby optimizing therapeutic regimens and reducing unnecessary treatment burden (Li et al., 2025).

Traditional clinical predictors of treatment response, such as baseline visual acuity or lesion type, provide limited prognostic value. In contrast, artificial intelligence models have demonstrated the ability to extract subtle imaging biomarkers from OCT and OCTA that correlate with treatment outcomes. Features such as retinal thickness dynamics, presence of subretinal or intraretinal fluid, hyperreflective foci, and pigment epithelial detachment have been incorporated into machine learning frameworks to predict short- and long-term response to anti-VEGF therapy. These models enable more refined stratification of patients, facilitating personalized treatment intervals and reducing overtreatment or undertreatment risks (Alryalat et al., 2022).

Yeh et al. (2022) addressed the challenge of predicting long-term visual outcomes in neovascular age-related macular degeneration (nAMD), where anti-VEGF injections remain the standard therapy but prognosis varies widely. They developed HDF-Net, a heterogeneous data fusion deep convolutional neural network (Wang et al., n.d.) capable of integrating pre-treatment OCT B-scans with demographic variables (age, gender, baseline visual acuity). By learning directly from both image and non-image data, the model bypasses the limitations of traditional machine-learning approaches that depend on hand-engineered OCT biomarkers. Trained and validated on 1,396 OCT image series from 698 patients, HDF-Net accurately predicted whether eyes would gain at least two lines of Snellen visual acuity 1 year after anti-VEGF therapy, achieving an AUC of 0.989 and accuracy of 93.6%, significantly outperforming conventional CNNs such as ResNet50 and AlexNet. Saliency (heat) maps confirmed that the algorithm focused on anatomically meaningful regions, including the foveal contour and ellipsoid zone, lending clinical plausibility to its predictions. By combining structural retinal information with simple baseline clinical metrics in a single end-to-end network, this study demonstrates the feasibility of personalized, data-driven treatment planning for nAMD. The approach can inform prognosis, optimize anti-VEGF injection schedules, and help set patient expectations, marking a substantive advance toward real-world precision medicine in retinal disease management.

Fu et al. (2021) leveraged a deep-learning segmentation algorithm to transform 137,379 OCT scans from a large real-world Moorfields Eye Hospital cohort into qOCT. After stringent selection, 926 treatment-naïve eyes with 12-month follow-up were analyzed to explore how these automatically derived metrics could predict functional outcomes during anti-VEGF therapy for nAMD. The study demonstrated that baseline qOCT biomarkers alone moderately predicted future visual acuity (*R*^2^ ≈ 0.15), but predictive accuracy improved markedly when baseline visual acuity (VA) and early treatment responses—both morphometric and functional—were incorporated. The best-performing multivariable models, combining qOCT data, baseline VA, and early VA change, achieved *R*^2^ up to 0.79 with a mean absolute error around 5 ETDRS letters for 12-month visual outcomes. In addition, the incremental VA gain from individual injections could be estimated, albeit with lower precision. Among the structural predictors, retinal pigment epithelium (RPE) volume was consistently associated with better visual prognosis, whereas subretinal hyperreflective material (SHRM) and intraretinal fluid (IRF) correlated with poorer outcomes. This work highlights the clinical potential of fully automated OCT biomarker extraction. By eliminating manual segmentation, the pipeline enables rapid, reproducible assessment of disease activity and early treatment response, paving the way for point-of-care decision-support systems. Such tools can help clinicians personalize anti-VEGF regimens, refine treat-and-extend strategies, and provide individualized prognostic counseling for patients with nAMD.

Han (2025) investigated whether deep learning can enhance management of nAMD, where anti-VEGF injections are the standard of care and OCT is the main imaging modality for monitoring disease activity. Using a DenseNet201 architecture, the study built two predictive models. The first model estimated the degree of anatomical improvement after intravitreal anti-VEGF injections. Trained on 2,068 OCT images from 517 Korean patients, and incorporating OCT scans taken both before and during the loading phase, the model achieved a sensitivity of 0.914 and an accuracy of 0.820, significantly outperforming ophthalmologists. The second model addressed an important follow-up challenge: forecasting disease recurrence within 3 months after confirming dry-up 1 month after three loading injections. Analyzing 1,076 OCT images from 269 patients, the algorithm achieved 53% accuracy from a single pre-injection scan and 60% accuracy when OCT images after each of the first three injections were included, again surpassing experienced specialists. These findings indicate that DenseNet201-based deep learning can reliably predict both treatment response and early recurrence in nAMD. Such predictive capability could guide individualized treat-and-extend protocols, optimizing injection frequency, reducing overtreatment, and ultimately improving patient outcomes.

Emerging evidence indicates that genetic factors contribute significantly to variability in therapeutic response. Variants in genes such as CFH, ARMS2/HTRA1, and VEGFA have been linked to differential efficacy of anti-VEGF agents. Integrating genetic profiles with imaging and clinical data through AI-driven multimodal models offers a powerful approach to predicting treatment outcomes at the individual level. For example, combining OCT-based fluid dynamics with complement gene variants may help identify patients who are less likely to respond to standard anti-VEGF therapy and who may benefit from alternative or adjunctive treatments, such as complement inhibitors or novel biologics.

By bridging imaging biomarkers, genetic predisposition, and clinical parameters, AI-based prediction models hold the potential to transform therapeutic decision-making in AMD. These tools can enable more precise tailoring of treatment regimens, reduce the economic and procedural burden of frequent injections, and ultimately improve visual outcomes in diverse patient populations.

### 3.3 AI-enabled longitudinal risk prediction and treatment optimization

Beyond early detection and treatment prediction, AI plays an increasingly important role in the longitudinal prognosis (Das et al., 2019) and monitoring of patients with AMD. Effective disease surveillance is critical given the chronic and progressive nature of AMD (Pawloff et al., 2023), as well as the substantial variability in individual disease trajectories.

AI algorithms trained on large, longitudinal imaging and clinical datasets have shown promise in forecasting functional outcomes (Wang et al., 2024b), particularly visual acuity decline. By analyzing temporal patterns in OCT-derived biomarkers—such as drusen volume, retinal thickness, and subretinal fluid dynamics—machine learning models can estimate the risk of vision loss over defined time horizons. Some models extend beyond structural features, incorporating multimodal data including demographics, comorbidities, and genetic information to generate personalized prognostic profiles. These predictive capabilities enable clinicians to identify high-risk patients who may benefit from closer monitoring, earlier intervention, or enrollment in clinical trials, while reducing unnecessary follow-up for those with more stable disease (Frank-Publig et al., 2025).

In parallel, AI has enhanced the development of home-based monitoring systems designed to extend clinical oversight into patients' daily lives. Smartphone-based applications and portable OCT devices allow patients to capture retinal images or perform functional tests such as visual distortion monitoring. AI-driven algorithms can automatically analyze these data to detect early signs of disease progression, such as conversion from dry to wet AMD or subtle changes in retinal morphology, and alert clinicians in real time. Combined with teleophthalmology platforms, these tools improve accessibility for patients in remote or underserved areas (Das et al., 2019) and reduce the burden of frequent in-person visits (Frank-Publig et al., 2025).

Das et al. (2019) introduced a cloud Internet of Medical Things (IoMT) teleophthalmology framework that integrates advanced deep learning with portable imaging to deliver scalable remote screening and follow-up for AMD. Central to the system is AMD-ResNet, a 152-layer residual convolutional neural network specifically optimized for four-stage AMD severity classification. Leveraging transfer learning from ImageNet and trained on more than 67,000 AREDS fundus images, AMD-ResNet achieved 97.5% accuracy, 94.9% sensitivity, and 98.3% specificity—significantly exceeding human grading performance. To address the need for longitudinal risk assessment, the study further proposed an LSTM-based progression model. Using temporal embeddings from AMD-ResNet, the LSTM architecture is designed to capture disease trajectory and forecast future severity stages, setting the stage for true precision medicine. A distinctive feature of the study is its end-to-end teleophthalmology design. The OphthoAI head-mounted fundus camera securely captures and transmits retinal images, while encrypted cloud infrastructure and an ophthalmologist dashboard enable seamless collaboration between AI algorithms and clinicians. This doctor-in-the-loop workflow supports continuous monitoring and interactive consultations, even in settings with intermittent internet connectivity. By combining cutting-edge deep learning (CNN + LSTM) with IoMT cloud infrastructure, Das et al. provide an integrated solution for large-scale, real-time AMD detection and progression prediction. The system is particularly well-suited for medically underserved regions, where early diagnosis and personalized follow-up can prevent avoidable blindness.

Pawloff et al. (2023) demonstrated that modern DL segmentation robustly detects and quantifies retinal fluid—matching specialist performance—across multiple OCT vendors. Crucially, central thickness measures (CSFT/CPT) correlate only modestly (IRF) to weakly (SRF) with true fluid burden, especially during treatment, and exhibit large residual errors. In practice, this supports shifting from thickness-based proxies to direct, automated IRF/SRF volume tracking for nAMD follow-up and treatment decisions.

Liu S. et al. (2025) conducted a retrospective single-center study using an improved LUNet deep-learning model to extract vascular biomarkers from OCT angiography (OCTA) in 165 patients with exudative age-related macular degeneration (nAMD) undergoing anti-VEGF therapy. Despite overall improvement in best-corrected visual acuity and central macular thickness after 6 months, 35.8% of patients showed inadequate response. Detailed imaging analysis revealed that greater macular neovascularization vessel dispersion (Vdisp-MNV) predicted better treatment outcomes, whereas larger neovascular surface area, the presence of pigment epithelial detachment, and lower deep vascular complex vessel dispersion (Vdisp-DVC) were associated with poorer response. Baseline Vdisp-DVC and intraretinal fluid further correlated with initial visual acuity. These findings demonstrate that OCTA-derived microvascular metrics can serve as early, objective indicators of therapeutic efficacy. By integrating automated LUNet-based OCTA analysis into routine care, clinicians could identify likely non-responders sooner, adjust dosing intervals, or adopt alternative regimens, enabling more precise, individualized management of nAMD and better allocation of clinical resources.

Collectively, AI-enabled prognostic modeling and remote monitoring represent a shift toward proactive and continuous care in AMD management. By integrating predictive analytics with patient-centered digital health solutions, these approaches hold the potential to preserve visual function more effectively while optimizing healthcare resources.

### 3.4 Explainable artificial intelligence in AMD precision therapeutics

Explainable artificial intelligence (XAI; Frank-Publig et al., 2025) has emerged as a critical component for the safe, transparent, and clinically acceptable deployment of AI systems in ophthalmology. Although deep learning algorithms have demonstrated remarkable performance in AMD screening, lesion segmentation, progression prediction, and treatment-response forecasting, many state-of-the-art models remain “black boxes (Parmar et al., 2025)” providing limited insight into how predictions are generated. In clinical practice, the lack of interpretability may reduce clinician trust, hinder regulatory approval, and limit adoption in real-world healthcare settings. Consequently, improving the explainability and transparency of AI systems has become increasingly important for precision therapeutics in AMD.

Several XAI techniques (Zhang et al., 2025) have been applied to retinal imaging analysis to improve model interpretability. Gradient-weighted Class Activation Mapping (Grad-CAM) and saliency mapping methods are commonly used to visualize retinal regions contributing most strongly to AI predictions. In AMD, these approaches can highlight clinically relevant pathological structures such as drusen, pigment epithelial detachment, geographic atrophy regions, subretinal fluid, intraretinal cysts, choroidal neovascularization, and hyperreflective foci on optical coherence tomography or fundus imaging. By visually correlating AI attention maps with known disease biomarkers, clinicians may better understand whether the algorithm is relying on biologically plausible features rather than spurious correlations.

In addition to visualization-based methods, feature attribution techniques such as SHapley Additive exPlanations (SHAP) and Local Interpretable Model-Agnostic Explanations (LIME) provide quantitative assessment of feature importance in predictive models. These approaches may help identify the relative contributions of imaging biomarkers, demographic characteristics, genetic variants, and clinical parameters to AMD progression risk or therapeutic response prediction. For example, explainable multimodal systems may reveal how retinal thickness, drusen burden, complement pathway polymorphisms, smoking history, and anti-VEGF injection frequency collectively influence disease prognosis. Such interpretability may not only improve clinician confidence but also facilitate biomarker discovery and hypothesis generation in translational ophthalmic research.

Attention-based architectures and transformer models further offer opportunities for interpretable multimodal learning in AMD precision medicine. Attention visualization can illustrate how AI systems dynamically prioritize specific retinal regions, imaging modalities, or longitudinal clinical variables during decision-making. In multimodal AMD frameworks integrating OCT, OCT angiography (Liu J. et al., 2025), fundus photography, genomics, and electronic health record data, interpretable fusion strategies may become particularly important for understanding cross-modal interactions and disease heterogeneity. These methods may support more individualized therapeutic stratification and longitudinal monitoring strategies (Katschke et al., 2025).

Another important dimension of explainable AI (He et al., 2025) involves uncertainty quantification and confidence estimation. In real-world clinical settings, AI systems inevitably encounter low-quality images, atypical disease presentations, device variability, and domain shift across healthcare institutions. Uncertainty-aware AI frameworks can identify cases where predictions are unreliable or require human review, thereby reducing the risk of unsafe automated recommendations. Bayesian deep learning, ensemble learning, Monte Carlo dropout, and calibration analysis have therefore gained increasing interest in ophthalmic AI research. Such approaches may be especially valuable in high-stakes therapeutic decisions, including anti-VEGF treatment interval optimization and progression prediction for geographic atrophy.

From a regulatory and ethical perspective, explainability is increasingly regarded as essential for trustworthy medical AI. Regulatory agencies and clinical governance frameworks are placing growing emphasis on transparency, fairness, reproducibility, and human oversight in AI-assisted healthcare systems. In AMD precision therapeutics, interpretable AI systems (Michl et al., 2025) may facilitate clinician-AI collaboration, improve patient communication, and support informed decision-making processes. Explainable outputs may also help clinicians verify whether AI predictions are consistent with established pathological mechanisms and clinical reasoning.

Despite these advances, significant challenges remain. Many existing explainability methods provide only *post hoc* interpretations and may not fully reflect the true internal reasoning processes of deep neural networks. Furthermore, standardized evaluation metrics for explainability remain underdeveloped, and the clinical utility of many XAI methods has not yet been prospectively validated. Future research should therefore focus on developing inherently interpretable architectures, clinically meaningful explanation frameworks, multimodal causal inference models, and standardized benchmarks for evaluating transparency and trustworthiness in ophthalmic AI systems.

Overall, explainable artificial intelligence represents a foundational component of next-generation AMD precision therapeutics. By improving transparency, clinician trust, biomarker interpretability, and safety monitoring, XAI may help bridge the gap between experimental AI research and real-world personalized ophthalmic care.

### 3.5 Panoramic view of algorithmic advances in AMD care

The body of evidence reviewed highlights significant methodological diversity and progressive clinical integration of AI in the detection, staging, and management of AMD.

**Methodological advances**. A range of deep-learning architectures has been applied to different data modalities. For early disease detection, Saha et al. demonstrated that CNNs with transfer learning can identify subtle spectral-domain OCT biomarkers—such as subretinal drusenoid deposits and hyper-reflective foci—with accuracies approaching 90%. Savoy et al. (2024) extended this paradigm to smartphone-based fundus photography, maintaining high area-under-curve (AUC) values (~0.95), while Ejaz et al. applied CNNs to multi-disease fundus classification with comparable accuracy. Multimodal integration of OCT, OCT angiography (OCTA), and structural B-scan data, as reported by Thakoor et al., achieved near-95% accuracy for three-class AMD staging, and Yeh et al. demonstrated that fusing OCT images with baseline clinical covariates markedly improves prediction of anti-VEGF treatment outcomes (AUC 0.989). Predictive modeling of treatment response has likewise advanced: Fu et al. (2021) combined automated quantitative OCT segmentation with machine-learning regression to forecast visual acuity up to 12 months (*R*^2^ up to 0.79), while Han (2025) and Liu S. et al. (2025) used DenseNet and an improved LUNet model, respectively, to predict anti-VEGF responsiveness and neovascular AMD recurrence from OCT and OCTA vascular features. Das et al. integrated a deep CNN (AMD-ResNet) with a long short-term memory (LSTM) network within a cloud-based Internet of Medical Things (IoMT; Mathkor et al., 2024) framework to enable remote, sequential prediction of AMD progression. In parallel, Pawloff et al. (2023) and Crincoli et al. (2024) focused on robust, device-agnostic fluid segmentation and risk stratification, and Li et al. introduced a non-invasive serum three-metabolite panel as a molecular diagnostic and staging tool.

**Clinical applications and translational relevance**. These AI approaches collectively cover the full continuum of AMD care. Screening and triage solutions (e.g., Savoy et al., 2024; Ejaz et al., 2024; Saha et al., 2019) offer scalable population-level detection, including in resource-limited settings. Intermediate-stage phenotyping and risk prediction (e.g., Crincoli et al., 2024; Frank-Publig et al., 2025; Liu S. et al., 2025) support tailored monitoring intervals and timely prophylactic interventions. Models targeting treatment-response forecasting and longitudinal monitoring (e.g., Fu et al., 2021; Han, 2025; Pawloff et al., 2023; Liu S. et al., 2025) enable personalized anti-VEGF regimens such as treat-and-extend protocols, early switching, or combination strategies. Cloud-based and IoMT-enabled architectures (e.g., Das et al., 2019) further extend reach to underserved populations while supporting continuous, ophthalmologist-in-the-loop oversight.

**Synthesis and implications**. Despite differences in datasets, imaging platforms, and algorithmic design, three converging themes emerge. First, precision improves with multimodality and automation: combining OCT structural and vascular data, clinical covariates, and molecular biomarkers consistently outperforms single-modality approaches. Second, there is a paradigm shift from detection to individualized management: contemporary models increasingly predict therapeutic demand, functional outcomes, or recurrence risk, thereby informing real-time clinical decisions. Third, scalability and equity are achievable: smartphone imaging, wearable devices, and cloud-based AI create opportunities for global deployment and more equitable access to specialist-level diagnostics.

Collectively, these studies indicate that AI in AMD is transitioning from an adjunctive image-analysis tool to a comprehensive precision-ophthalmology infrastructure, capable of early detection, dynamic disease monitoring, and personalized treatment planning. Such integration of multimodal deep learning and molecular data promises to improve outcomes, reduce treatment burden, and enhance the efficiency of ophthalmic care delivery worldwide.

### 3.6 Limitations and translational challenges of AI in AMD

Despite the remarkable progress achieved by artificial intelligence systems in AMD diagnosis, prognosis, and therapeutic prediction, substantial translational challenges remain before these technologies can be safely and effectively integrated into routine clinical practice. Although many studies report high diagnostic accuracy and promising predictive performance, the majority of current AI models remain at an early developmental stage and have not yet demonstrated robust real-world clinical utility.

One major limitation of existing AMD AI research is the widespread reliance on retrospective single-center datasets. Many published studies are developed using relatively small datasets collected from highly specific patient populations or individual healthcare institutions, which may not adequately represent the diversity of real-world clinical environments. Limited demographic variation in age, ethnicity, disease subtype, and imaging characteristics may increase the risk of overfitting and reduce the generalizability of AI systems across broader patient populations. Furthermore, the prevalence of class imbalance in ophthalmic datasets, particularly for rare disease stages or treatment outcomes, may artificially inflate performance metrics while masking poor sensitivity in clinically important subgroups.

Another important challenge involves heterogeneity in ophthalmic imaging acquisition and annotation. Variability across imaging devices, manufacturers, acquisition protocols, image quality, and segmentation standards may substantially influence algorithmic performance. Domain shift between institutions and imaging platforms remains a significant obstacle for external deployment, particularly for optical coherence tomography and OCT angiography-based AI systems. In addition, annotation quality often depends on expert interpretation, which may introduce interobserver variability and labeling inconsistency. The lack of universally standardized imaging datasets and annotation frameworks further complicates direct comparison between studies.

A critical methodological concern is that many AMD AI studies rely primarily on internal validation, while prospective multicenter validation and external testing remain relatively limited. Internal validation alone may overestimate real-world performance because training and testing data are often derived from similar distributions. Potential issues such as data leakage, spectrum bias, selection bias, and inadequate calibration analysis may further contribute to inflated accuracy estimates. Importantly, strong performance on retrospective benchmark datasets does not necessarily guarantee reliable performance in real-world clinical workflows. Future studies should therefore emphasize multicenter prospective trials, independent external validation, and clinically meaningful outcome evaluation.

Explainability and transparency also represent major barriers to clinical adoption. Many high-performing deep learning models operate as “black-box” systems, providing limited insight into the reasoning processes underlying algorithmic predictions. In ophthalmology, clinicians must be able to understand whether AI systems are relying on biologically plausible retinal biomarkers rather than spurious image artifacts or dataset-specific correlations. The absence of interpretable decision-making may reduce clinician trust, complicate regulatory approval, and hinder integration into patient care. Consequently, explainable artificial intelligence, uncertainty quantification, and confidence-aware prediction frameworks are increasingly recognized as essential components of trustworthy ophthalmic AI systems.

Another important translational limitation concerns the gap between algorithmic performance and actual clinical benefit. Improvements in image classification accuracy do not automatically translate into improved patient outcomes, treatment optimization, or healthcare efficiency. Many AI studies focus primarily on technical performance metrics such as accuracy, sensitivity, specificity, and area under the receiver operating characteristic curve (AUC), while comparatively fewer studies evaluate longitudinal patient outcomes, cost-effectiveness, workflow integration, clinician-AI interaction, or patient-centered benefits. The clinical utility of AI systems must therefore be evaluated not only through diagnostic performance but also through their ability to improve therapeutic decision-making, reduce disease burden, and enhance healthcare accessibility.

Ethical, legal, and regulatory considerations further complicate the implementation of AI-enabled precision therapeutics in AMD. Concerns regarding data privacy, algorithmic fairness, informed consent, cybersecurity, and accountability remain incompletely addressed. Bias arising from underrepresentation of certain ethnic or socioeconomic populations may contribute to healthcare inequities if AI systems are deployed without adequate fairness evaluation. Moreover, evolving regulatory frameworks for adaptive AI systems and continuously learning algorithms present additional challenges for long-term clinical deployment and monitoring.

Future progress in AI-enabled precision therapeutics for AMD will require collaborative efforts across clinicians, imaging scientists, bioinformaticians, regulatory agencies, and healthcare systems. Prospective multicenter studies, federated learning strategies, standardized benchmarking datasets, uncertainty-aware AI frameworks, and interpretable multimodal models may help improve robustness and clinical trustworthiness. In addition, integrating AI systems into clinician-centered workflows rather than fully autonomous decision-making paradigms may facilitate safer and more effective implementation. Ultimately, overcoming these translational barriers will be essential for realizing the full potential of AI-driven personalized ophthalmic care in AMD.

## 4 Precision therapeutics in AMD

Precision therapeutics in AMD should be considered as a continuum extending from optimization of currently available treatments to the development of next-generation targeted interventions. Although emerging approaches such as gene therapy, complement modulation, and regenerative stem cell-based therapies represent important advances toward biologically targeted treatment, precision therapeutics is not limited to the discovery of novel therapeutic agents. In clinical practice, a substantial proportion of precision therapeutic benefit may be achieved through individualized application of existing therapies by incorporating patient-specific imaging biomarkers, genetic susceptibility profiles, molecular characteristics, and longitudinal clinical trajectories. For example, AI-enabled prediction of anti-VEGF treatment response, recurrence risk, and optimal treatment intervals may facilitate personalized treatment selection and adaptive therapeutic strategies for neovascular AMD. Therefore, AI serves not only as a tool for identifying future therapeutic targets but also as an enabling technology for improving the effectiveness, efficiency, and personalization of currently established AMD treatments.

### 4.1 Current precision treatment approaches for AMD

The therapeutic landscape of AMD has evolved substantially over the past two decades, with increasing emphasis on precision strategies that account for inter-individual variability in disease mechanisms and treatment response.

Intravitreal anti-VEGF therapy remains the cornerstone of treatment for neovascular (wet) AMD (Marchesi et al., 2024). Agents such as ranibizumab, aflibercept, and, more recently, brolucizumab have demonstrated significant efficacy in reducing choroidal neovascular activity, stabilizing disease, and improving visual outcomes. Despite these advances, treatment response is highly variable. A majority of patients achieve stabilization or modest improvement in vision, yet approximately one-third demonstrate incomplete or poor response, with persistent fluid or progressive scarring despite intensive therapy. Furthermore, the necessity for repeated intravitreal injections imposes a considerable clinical and economic burden, underscoring the need for individualized treatment regimens. Current research is increasingly focused on identifying imaging and molecular biomarkers—such as retinal thickness dynamics or genetic variants in CFH and ARMS2/HTRA1—that may predict therapeutic responsiveness and guide personalized dosing schedules (Mishra et al., 2025).

For geographic atrophy associated with advanced dry AMD, effective therapies remain limited. However, complement pathway inhibition has emerged as a promising avenue given the strong genetic and molecular evidence implicating complement dysregulation in AMD pathogenesis. Recent phase III trials have demonstrated that intravitreal inhibitors targeting complement components, such as C3 (pegcetacoplan) and C5 (avacincaptad pegol), can slow the progression of GA, marking a critical advance in addressing an area of significant unmet need. Beyond complement inhibition, novel biologics are being explored, including agents targeting oxidative stress, mitochondrial dysfunction, and angiogenesis beyond VEGF signaling. Gene therapy approaches and sustained-release drug delivery platforms further exemplify efforts to enhance treatment durability and precision.

Thus, these developments reflect a paradigm shift toward precision therapeutics in AMD (Zhang et al., 2024), where treatment selection and dosing strategies are increasingly guided by patient-specific characteristics. This individualized approach not only aims to maximize efficacy but also to minimize treatment burden, improve cost-effectiveness, and expand therapeutic options for both wet and dry forms of AMD.

### 4.2 Gene-based stratification of AMD therapies

The identification of genetic susceptibility loci in AMD has provided important opportunities to move beyond a “one-size-fits-all” treatment paradigm toward gene-based therapeutic stratification (Hushmandi et al., 2025). By linking genetic profiles with clinical outcomes, researchers aim to personalize interventions that are tailored to the molecular underpinnings of an individual's disease.

Variants in complement-related genes, most notably CFH, C3, and CFI, influence the degree of complement dysregulation in the macula and may help identify patients more likely to benefit from complement inhibitors (Finocchio et al., 2023). Similarly, allelic variants in the ARMS2/HTRA1 locus, which are associated with angiogenesis and extracellular matrix remodeling, have been investigated as modulators of responsiveness to anti-VEGF therapy. Incorporating such genetic insights into clinical decision-making frameworks may enable a more precise allocation of patients to therapeutic classes that target their predominant pathogenic pathway, whether complement-mediated inflammation, lipid dysregulation, or angiogenesis.

Several studies have demonstrated associations between specific genetic polymorphisms and treatment outcomes (Akyol and Lotery, 2020). For example, CFH Y402H polymorphisms have been correlated with reduced responsiveness to anti-VEGF agents, suggesting that patients carrying high-risk alleles may require closer monitoring or alternative adjunctive therapies. Likewise, ARMS2/HTRA1 risk variants have been linked to more aggressive disease progression and variable treatment outcomes, potentially guiding intensity of anti-VEGF regimens (Cui, 2024). In addition, emerging evidence implicates polymorphisms in VEGFA itself as modifiers of therapeutic efficacy, highlighting the relevance of drug-target gene interactions. Although many of these associations remain preliminary, they illustrate the potential of pharmacogenomics to refine therapeutic strategies.

By integrating genetic stratification with imaging biomarkers and artificial intelligence frameworks, the field is progressing toward a comprehensive model of precision therapeutics in AMD. Such approaches could enable clinicians to identify likely responders, select optimal drug classes, and personalize treatment frequency, thereby improving clinical outcomes while reducing treatment burden.

### 4.3 Stem cell therapy for AMD

Stem cell-based therapies have emerged as a promising avenue for the treatment of AMD, particularly in cases of advanced dry AMD and geographic atrophy, where conventional pharmacologic interventions remain inadequate (Giacalone et al., 2024). The rationale for stem cell therapy lies in the potential to restore or replace degenerated RPE cells and photoreceptors, thereby re-establishing the structural and functional integrity of the macula.

Several stem cell types have been investigated for AMD therapy (Dehghan et al., 2022). Human embryonic stem cells (hESCs) and induced pluripotent stem cells (iPSCs) can be differentiated into RPE-like cells for subretinal transplantation, offering the potential to replace dysfunctional RPE and support photoreceptor survival. Mesenchymal stem cells (MSCs), derived from bone marrow, adipose tissue, or umbilical cord, have also been evaluated, largely due to their paracrine effects, including immunomodulation, angiogenesis regulation, and neuroprotection. Early-phase clinical trials have demonstrated the feasibility and relative safety of these approaches, with reports of anatomical integration and modest functional improvements in some patients.

Two main strategies are currently under evaluation: (1) suspension injections of stem cell-derived RPE cells into the subretinal space, and (2) transplantation of RPE monolayers on scaffolds designed to mimic Bruch's membrane. The latter approach offers improved cell survival and alignment but poses greater surgical complexity. Advances in biomaterials and surgical techniques continue to refine these delivery platforms, aiming to optimize graft survival, integration, and long-term functional benefit (Liu et al., 2024).

Kiliç et al. (2025) demonstrated in an *in-vitro* AMD model that combining curcumin with mitochondria transferred from human Wharton's Jelly- or endometrium-derived mesenchymal stem cells (hWJ-MSC-mt, hE-MSC-mt) protects retinal pigment epithelial (ARPE-19) cells against oxidative stress. Curcumin improved cell survival and suppressed oxidative and inflammatory markers, while mitochondrial transfer enhanced mitochondrial function (e.g., TOMM20) and retinal health genes (RPE65, RLBP1) and reduced damage-related genes (HTRA1, ARMS2). Synergistic effects were most evident when curcumin was combined with hWJ-MSC-mt or hE-MSC-mt, supporting their potential as a novel, mitochondria-targeted therapeutic strategy for AMD, pending *in-vivo* and clinical validation.

Despite encouraging progress, significant challenges remain in translating stem cell therapy into routine clinical practice (Wang et al., 2025). Risks of tumorigenicity, immune rejection, and variable differentiation efficiency must be carefully managed. Standardization of cell preparation protocols and long-term safety monitoring are essential. Moreover, patient stratification will likely be critical, as stem cell therapy may be most beneficial in earlier stages of RPE dysfunction before extensive photoreceptor loss occurs. Integration of stem cell therapy with precision medicine approaches—such as combining genetic risk stratification, imaging biomarkers, and AI-driven prognosis—could help identify patients most likely to benefit and improve treatment outcomes.

In summary, stem cell therapy represents a frontier in the management of AMD, offering the potential not only to halt disease progression but also to restore lost retinal function. While still experimental, ongoing advances in cell biology, biomaterials, and regenerative medicine are steadily paving the way for its clinical translation.

### 4.4 Challenges in AMD precision therapeutics

While the integration of genetic insights, molecular profiling, and artificial intelligence offers a compelling framework for precision therapeutics in AMD, several challenges continue to impede clinical translation and widespread adoption.

AMD is a multifactorial disease influenced by genetic susceptibility, environmental exposures, systemic comorbidities, and stochastic biological processes. Although susceptibility genes such as CFH, ARMS2/HTRA1, and C3 have been implicated in disease mechanisms, their effect sizes are modest, and gene-gene as well as gene-environment interactions remain incompletely understood. This heterogeneity complicates efforts to stratify patients reliably and necessitates integrative models that account for multiple dimensions of risk.

Although imaging biomarkers (e.g., drusen volume, subretinal fluid dynamics) and genetic variants have shown promise in predicting disease course or treatment response, reproducibility across diverse populations and clinical settings is limited. Many AI-driven models are trained on relatively homogenous datasets, raising concerns regarding their generalizability to broader patient cohorts. Moreover, the absence of standardized criteria for biomarker validation hinders regulatory approval and clinical implementation.

Current precision therapies remain constrained by incomplete efficacy. Anti-VEGF agents, while highly effective for many, fail to benefit a substantial subset of patients. Complement inhibitors have demonstrated modest reductions in the progression of geographic atrophy but do not fully restore function or halt disease progression. Stem cell therapies and gene-based interventions remain experimental, with unresolved issues of long-term safety, durability, and scalability.

The adoption of precision therapeutics is further challenged by logistical and economic considerations. Routine integration of genetic testing into clinical workflows is not yet standard practice, partly due to cost, lack of reimbursement, and limited clinician familiarity with interpreting genomic data. Similarly, AI systems require extensive validation, clinician training, and integration into electronic health record systems before they can be effectively deployed in routine care.

The increasing reliance on genetic profiling and AI raises concerns regarding patient privacy, informed consent, and data security. Moreover, inequities in access to advanced diagnostics and personalized therapies risk exacerbating healthcare disparities, particularly in low-resource settings. Regulatory frameworks must evolve to address these ethical concerns while ensuring the safety and efficacy of emerging precision interventions.

In summary, although precision therapeutics holds great promise for transforming AMD management, substantial biological, technological, clinical, and ethical challenges remain. Overcoming these barriers will require interdisciplinary collaboration, robust multicenter validation studies, and the development of standardized frameworks to ensure that precision approaches are both scientifically rigorous and clinically feasible.

## 5 Integration of AI and precision therapeutics

The convergence of AI and precision therapeutics represents a transformative paradigm in the management of AMD. By leveraging advanced computational methods to integrate multimodal datasets—including imaging, genetic, molecular, and clinical information—AI provides the analytical framework (Wang, 2025b) required to operationalize precision medicine in real-world clinical practice.

### 5.1 Multimodal data integration

AI systems excel at synthesizing heterogeneous sources of information that reflect the multifactorial nature of AMD (Wang et al., 2024a). For example, combining high-resolution imaging biomarkers from regular fundus, OCT, fluorescein angiography fundus (Wang et al., 2023), ultra-widefield fundus images (Wang et al., n.d.) and OCTA with genetic variants in CFH or ARMS2/HTRA1 and clinical parameters such as age, lifestyle factors, and comorbidities allows for the construction of comprehensive patient-specific risk profiles. Machine learning algorithms can uncover latent patterns within these datasets that are not readily identifiable through conventional analysis, thereby facilitating more accurate diagnosis, risk stratification, and therapeutic decision-making (Iida et al., 2025).

### 5.2 Personalized therapy selection and optimization

AI-driven predictive models can identify patients most likely to respond to specific interventions, such as anti-VEGF therapy, complement inhibitors, or emerging biologics. By aligning genetic susceptibility with imaging phenotypes, these tools can inform the selection of optimal treatment strategies and dosing regimens. For example, patients carrying high-risk CFH variants with evidence of complement dysregulation on imaging may be prioritized for complement-targeted therapies, whereas those with ARMS2/HTRA1 risk alleles and aggressive neovascularization could benefit from more intensive anti-VEGF regimens (Mondal et al., 2025). This stratified approach holds the potential to improve treatment efficacy, reduce unnecessary interventions, and lower healthcare costs.

### 5.3 Dynamic prognosis and monitoring

AI-enabled systems can also support continuous disease monitoring and adaptive therapeutic planning. Longitudinal analyses of OCT data (Li et al., 2020), coupled with home-based monitoring and teleophthalmology platforms, enable dynamic prediction of disease trajectories. Integration with genetic and molecular profiles further refines these predictions, ensuring that therapeutic strategies remain responsive to changes in individual risk over time.

### 5.4 Toward a learning healthcare system

The integration of AI and precision therapeutics aligns with the concept of a learning healthcare system (Golburean et al., 2024), in which real-world clinical data continuously contribute to the refinement of predictive models and therapeutic strategies. In AMD management, emerging large language models [54] and multimodal foundation models (Elgendy et al., 2026) may provide supportive functions such as automated clinical documentation, ophthalmic report summarization, and longitudinal data organization within teleophthalmology and electronic health record environments.

However, the clinical application of these systems in AMD precision therapeutics remains preliminary. Current evidence supporting autonomous diagnostic or therapeutic reasoning by large language models is limited, and important challenges—including hallucination, explainability, reliability, data privacy, and regulatory oversight—remain unresolved. Consequently, these technologies should presently be regarded as supportive and investigational tools rather than independent clinical decision-making systems. Future progress will require rigorous prospective validation, clinician-centered integration, and careful regulatory evaluation prior to routine clinical implementation.

### 5.5 Pipelines of patient-centered and clinician-centered pathways

Effective integration of AI into precision therapeutics requires parallel pipelines tailored to both patients and clinicians (Mathkor et al., 2024). For patients, AI facilitates engagement and empowerment by transforming multimodal data into comprehensible risk profiles and actionable recommendations. Imaging from clinic-based OCT or home-monitoring devices, combined with genetic and lifestyle information, can be automatically processed to generate personalized risk assessments. These insights may be delivered through mobile health platforms, offering alerts about disease progression, reminders for follow-up, and lifestyle modifications aligned with genetic susceptibility. By providing individualized feedback, such systems foster proactive disease management, improve adherence to monitoring, and reduce delays in accessing care.

For clinicians, AI functions as an advanced decision-support system, integrating imaging, genomic, and clinical data to optimize therapeutic strategies. Predictive models can stratify patients according to their likelihood of responding to anti-VEGF agents, complement inhibitors, or experimental biologics, thereby enabling precise allocation of therapies. Integration with electronic health records allows AI-driven recommendations—such as adaptive dosing intervals or alerts for insufficient response—to be seamlessly incorporated into clinical workflows. Moreover, AI enhances longitudinal prognosis, supporting adaptive treatment adjustments based on disease trajectory rather than static clinical benchmarks. Together, these patient- and clinician-centered pipelines create a collaborative ecosystem in which AI not only augments clinical decision-making but also empowers patients to participate actively in their care, thereby advancing the realization of precision therapeutics in AMD.

**Figure 2:**
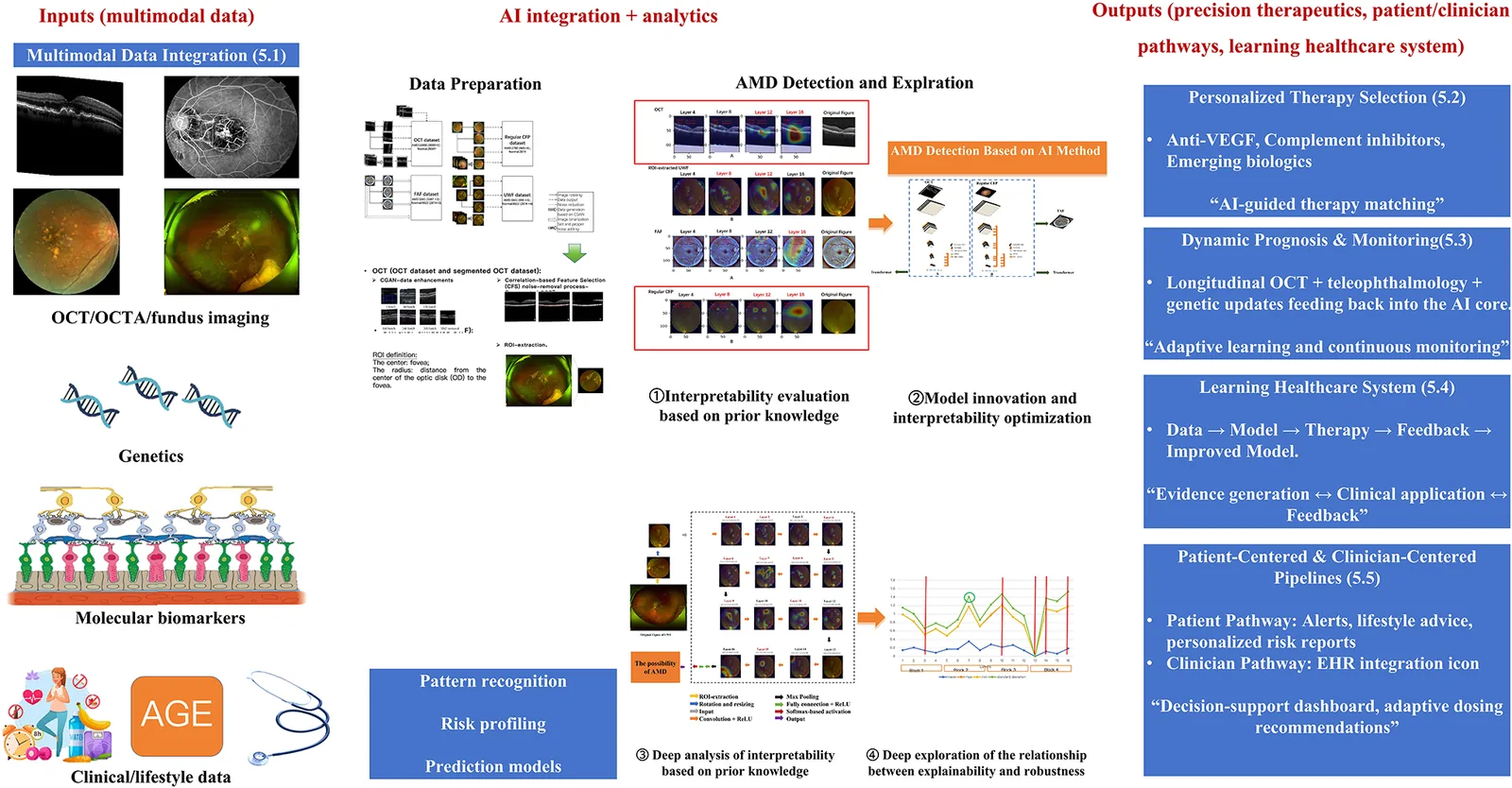
Integrative framework of artificial intelligence and precision therapeutics in AMD management.

Figure 2 illustrates the integrative framework combining AI and precision therapeutics for AMD (Wang, 2025a). On the left, multimodal data inputs—including OCT/OCTA/fundus imaging, genetic variants, molecular biomarkers, and clinical/lifestyle information—are collected. These heterogeneous datasets converge within the AI integration core, where machine learning models perform pattern recognition, risk profiling, and predictive analytics. The outputs (right) demonstrate how AI-driven insights enable precision therapeutics, spanning personalized therapy selection, dynamic disease monitoring, and adaptive treatment optimization. The framework supports the concept of a learning healthcare system, in which real-world data continually refine predictive models. Two distinct yet connected pipelines are highlighted: patient-centered pathways that empower individuals through mobile health platforms and risk assessments, and clinician-centered pathways that provide advanced decision-support tools seamlessly integrated with electronic health records. Together, this ecosystem shifts AMD management from reactive treatment toward proactive, individualized care.

In summary, the integration of AI with precision therapeutics has the potential to shift AMD management from reactive treatment to proactive, individualized care. By uniting computational analytics with molecular and clinical insights, this paradigm directly addresses the heterogeneity of AMD and establishes a scalable framework for personalized medicine in ophthalmology.

## 6 Clinical interpretation and translational application of AI in AMD precision therapeutics

Following the framework introduced in Section 1.3, current AI applications in AMD precision therapeutics can be interpreted according to different levels of clinical maturity and translational readiness. The framework does not rank AI technologies solely according to algorithmic performance; instead, it evaluates their potential clinical impact considering evidence strength, validation status, interpretability, workflow integration, and real-world applicability (von der Emde et al., 2026; Chen et al., 2026a). The following sections discuss representative characteristics, opportunities, and limitations associated with each translational level.

### 6.1 Level I: near-reference-standard AI systems

Level I AI systems represent the most clinically mature category within the proposed clinical readiness framework for AMD precision therapeutics (Saha et al., 2019). These applications primarily involve imaging-based AI models that demonstrate strong reproducibility, relatively standardized analytical workflows, and high agreement with retinal specialists under controlled clinical settings. In many cases, these systems function as objective quantitative measurement tools capable of reducing interobserver variability and improving consistency in longitudinal disease assessment. Consequently, certain Level I applications may approach a near-reference-standard role in specific ophthalmic imaging tasks.

The development of Level I AI systems has been facilitated by advances in high-resolution ophthalmic imaging and the increasing availability of annotated retinal datasets (Chen et al., 2026b). Typical evidence sources include fundus photography, OCT, and OCTA, all of which provide relatively structured and quantifiable imaging biomarkers (Li et al., 2026) suitable for deep learning analysis (Thakoor et al., 2022). Compared with more exploratory multimodal precision medicine approaches, these imaging modalities generally offer greater standardization and clearer biological interpretability, thereby supporting more reliable AI performance.

Representative clinical applications within this category include geographic atrophy segmentation, retinal fluid quantification, drusen measurement, CNV detection, and automated AMD staging (Chen et al., 2023). Among these tasks, AI-based retinal fluid quantification and geographic atrophy segmentation have demonstrated particularly strong translational potential because they involve objective anatomical features that can be repeatedly measured across longitudinal follow-up examinations. Automated segmentation systems may also improve workflow efficiency by reducing manual grading burden and enhancing monitoring consistency during anti-VEGF therapy.

From a translational perspective, Level I AI systems currently exhibit the highest degree of clinical maturity among AMD-related AI technologies (Pawloff et al., 2023). Several models have undergone external validation and demonstrated performance comparable to expert graders in specific imaging tasks. These systems may therefore contribute to more standardized disease monitoring, objective biomarker quantification, and scalable ophthalmic screening workflows. Furthermore, the relatively interpretable nature of imaging-based outputs may facilitate clinician trust and support regulatory evaluation compared with more opaque multimodal predictive systems.

Despite these advances, important limitations remain. The performance of Level I AI systems continues to depend heavily on image quality, imaging protocol consistency, and device-specific characteristics (Wang et al., 2023). Domain shift across institutions, imaging platforms, and patient populations may still substantially affect model robustness and generalizability. In addition, regulatory approval pathways, clinical workflow integration, interoperability with electronic healthcare systems, and real-world deployment logistics remain ongoing translational challenges. Therefore, although Level I AI applications represent some of the most clinically advanced forms of AI in AMD management, continued prospective validation and implementation-focused research remain essential prior to widespread autonomous clinical deployment.

### 6.2 Level II: advanced clinical decision-support AI

Level II AI systems represent one of the most clinically promising categories within the current landscape of AMD precision therapeutics (Crincoli et al., 2024). Unlike Level I applications that primarily function as objective imaging-based analytical tools, Level II systems are designed to support complex clinical decision-making processes, including therapeutic stratification, personalized monitoring, and longitudinal disease management. These AI applications do not operate as fully autonomous systems but instead function as advanced clinical decision-support tools that assist ophthalmologists in optimizing individualized patient care under physician supervision.

A defining characteristic of Level II AI systems is their ability to integrate longitudinal imaging information, treatment history, and clinical metadata to generate predictive insights relevant to therapeutic management. Common evidence sources include OCT, OCTA, serial longitudinal retinal imaging, demographic and clinical variables, and anti-VEGF treatment records (Fu et al., 2021). Compared with purely imaging-based segmentation systems, these models often involve more complex temporal and multimodal predictive tasks (Han, 2025) requiring interpretation of dynamic disease progression patterns over time.

Representative applications within this category include anti-VEGF response prediction, injection interval optimization, disease progression forecasting, conversion risk prediction from dry AMD to nAMD, and personalized monitoring schedule generation. Among these, AI-assisted anti-VEGF response prediction has emerged as a particularly important translational application because of the substantial interpatient variability in therapeutic response and treatment burden associated with chronic intravitreal injection therapy. By identifying imaging biomarkers, fluid recurrence patterns, or clinical characteristics associated with treatment response, AI systems may help optimize retreatment strategies and improve personalized disease management.

From a translational perspective, Level II AI systems currently demonstrate some of the highest potential clinical utility in AMD care. These approaches may improve workflow efficiency, reduce unnecessary treatment burden, facilitate earlier intervention, and support individualized monitoring strategies (Wang et al., n.d.). In addition, clinician-assisted AI systems may enhance decision consistency while maintaining essential human oversight and clinical judgment. Consequently, this category likely represents the most immediately impactful translational direction for AI-enabled precision therapeutics in ophthalmology (Wang, 2025a).

Despite these promising advances, important limitations remain. Most Level II AI systems continue to rely predominantly on retrospective datasets, and prospective multicenter validation remains relatively limited. Variability in imaging protocols, treatment regimens, follow-up intervals, and patient populations may substantially affect model robustness and generalizability across healthcare systems. Furthermore, temporal prediction models may be particularly vulnerable to data leakage, hidden confounding variables, and bias introduced by non-standardized treatment patterns. The interpretability of therapeutic prediction models also remains an ongoing challenge (Miladinović et al., 2026), especially when AI recommendations influence longitudinal treatment decisions.

Accordingly, although Level II systems demonstrate strong translational potential, widespread clinical deployment will require additional prospective validation, explainability enhancement, uncertainty-aware prediction frameworks, and careful integration into clinician-centered workflows. Maintaining physician oversight remains essential to ensure safe and reliable implementation of AI-assisted therapeutic decision support in AMD precision medicine.

### 6.3 Level III: emerging precision therapeutic AI

Level III AI systems represent emerging investigational approaches aimed at advancing precision therapeutics and individualized ophthalmic care in AMD (Li et al., 2025). Unlike more mature imaging-based systems, these models seek to integrate heterogeneous multimodal data sources—including genomics, molecular biomarkers, multimodal retinal imaging, and longitudinal clinical information—to support future precision medicine strategies. Although these approaches remain at an early translational stage, they are biologically promising and may eventually enable highly individualized therapeutic prediction, disease trajectory modeling, and biomarker-guided intervention.

A major characteristic of Level III systems is their exploratory and multimodal nature. These AI frameworks frequently combine data derived from genomics, proteomics, transcriptomics, molecular biomarkers (Chen et al., 2026b), wearable monitoring systems, and advanced ophthalmic imaging modalities such as OCT, OCTA, and fundus photography (Lee et al., 2026). By integrating these diverse data streams, Level III AI aims to move beyond traditional imaging-based disease classification toward more comprehensive biological characterization of AMD heterogeneity and therapeutic response variability.

Representative applications within this category include genotype-phenotype prediction, complement inhibitor response forecasting, stem cell therapeutic stratification, multimodal progression prediction, and personalized disease trajectory modeling. For example, multimodal AI systems may potentially identify molecular or imaging biomarkers associated with responsiveness to complement-targeted therapies or anti-VEGF treatment. Similarly, integrated genomic and imaging models may help characterize distinct AMD subtypes and support future precision therapeutic selection strategies. Emerging approaches incorporating longitudinal multimodal data may also facilitate dynamic prediction of disease progression and individualized monitoring plans.

From a translational perspective, Level III AI systems offer several important opportunities for the future development of precision ophthalmology. These approaches may contribute to individualized therapeutic optimization, multimodal biomarker discovery, early identification of high-risk disease phenotypes, and more biologically informed disease stratification.

Furthermore, multimodal AI may help uncover previously unrecognized interactions between retinal imaging biomarkers, genetic susceptibility, inflammatory pathways, and therapeutic response patterns. Such capabilities could eventually support more personalized and mechanism-driven AMD management strategies.

Despite these promising possibilities, substantial limitations currently restrict the clinical applicability of Level III AI systems. Many studies rely on relatively small and highly specialized datasets with limited demographic diversity, increasing the risk of overfitting and poor generalizability. In addition, multimodal data integration remains technically challenging because of differences in data structure, temporal synchronization, annotation quality, and biological interpretability across modalities. Standardized multimodal datasets and harmonized analytical protocols remain limited, further complicating reproducibility and cross-study comparison.

Another major limitation is the lack of large-scale prospective validation. Most Level III AI approaches remain investigational and have not yet undergone rigorous multicenter clinical evaluation. The complexity of integrating genomics, proteomics, molecular biomarkers, and longitudinal imaging data also introduces substantial computational and interpretability challenges. Consequently, although these systems demonstrate strong theoretical potential, they currently remain at an early translational stage and should be interpreted cautiously until supported by larger prospective studies and clinically validated implementation frameworks.

Nevertheless, Level III AI systems represent an important future direction in AMD precision therapeutics. As multimodal datasets become larger, more standardized, and increasingly integrated with explainable AI and longitudinal clinical monitoring frameworks, these investigational approaches may eventually contribute to the development of more precise, biologically informed, and individualized ophthalmic care.

### 6.4 Level IV: supportive and supplementary AI systems

Level IV AI systems primarily function as supportive and supplementary technologies designed to improve workflow efficiency, administrative coordination, communication, and patient engagement rather than directly determining therapeutic decisions in AMD (Ejaz et al., 2024). Unlike higher-level clinical decision-support systems, these applications are not intended to independently guide diagnosis or treatment selection. Instead, they serve as auxiliary tools that assist clinicians, healthcare systems, and patients by optimizing non-primary clinical processes surrounding AMD management.

A defining characteristic of Level IV systems is their workflow-oriented and supportive role. These AI applications typically provide administrative assistance, automated communication, documentation support, and teleophthalmology facilitation while maintaining relatively low direct therapeutic authority. Most systems within this category are designed to augment healthcare delivery efficiency rather than replace physician judgment. Consequently, they are generally considered lower-risk implementations compared with autonomous diagnostic or therapeutic AI systems.

Typical evidence sources for Level IV applications include electronic health records (EHRs), clinical text data, patient questionnaires, conversational systems, and healthcare workflow information. Advances in natural language processing (NLP) and LLMs, including GPT-based architectures, have contributed to the rapid development of AI-assisted clinical communication and documentation systems (Neri et al., 2026). These technologies may facilitate automated extraction, summarization, and organization of clinical information relevant to AMD management.

Representative applications include GPT-assisted clinical documentation, patient education chatbots, automated follow-up systems, teleophthalmology support platforms, and AI-assisted clinical report generation. For example, conversational AI systems may help provide patient education regarding anti-VEGF therapy schedules, medication adherence, or symptom monitoring. Similarly, automated reminder systems may improve follow-up compliance and facilitate longitudinal monitoring in elderly AMD populations requiring frequent clinic visits. AI-assisted documentation systems may also help reduce administrative burden by generating preliminary clinical summaries or organizing ophthalmic imaging reports.

From a clinical perspective, Level IV AI systems may provide several practical benefits. These technologies have the potential to improve workflow efficiency, reduce clinician workload, streamline documentation processes, and enhance patient accessibility to ophthalmic care services. In teleophthalmology settings, supportive AI systems may facilitate remote communication, triage coordination, and longitudinal patient engagement, particularly in resource-limited or geographically underserved regions. Consequently, although these systems do not directly determine therapeutic management, they may still contribute indirectly to improved healthcare delivery and patient experience.

Nevertheless, important limitations remain. Level IV systems should not independently determine diagnostic conclusions or therapeutic decisions because their outputs may lack sufficient clinical reliability, contextual understanding, or ophthalmic specificity. Large language model-based systems, in particular, remain vulnerable to hallucination, inaccurate summarization, incomplete reasoning, and generation of non-evidence-based recommendations. Furthermore, concerns related to patient privacy, cybersecurity, regulatory oversight, and clinical accountability remain incompletely resolved. Therefore, continued physician oversight and careful validation remain essential when integrating supportive AI systems into ophthalmic workflows.

Overall, Level IV AI applications represent an important auxiliary component of future ophthalmic healthcare systems. While these technologies currently function primarily as supportive tools rather than primary clinical decision-making systems, they may play a valuable role in improving workflow efficiency, patient engagement, and healthcare accessibility within evolving AI-assisted AMD management frameworks.

### 6.5 Level V: limited or currently unsuitable AI applications

Level V AI systems represent applications that currently lack sufficient evidence, reproducibility, interpretability, or clinical reliability to support safe implementation in AMD management. Unlike higher-level categories that demonstrate varying degrees of translational readiness, Level V approaches remain predominantly exploratory and may carry substantial risks if prematurely integrated into real-world clinical workflows. This category is particularly important for distinguishing scientifically promising concepts from overhyped or weakly validated technologies that currently lack adequate clinical support (Kim et al., 2026).

A defining characteristic of Level V systems is the combination of insufficient validation, unstable performance, poor generalizability, and limited biological interpretability. Many approaches within this category are supported primarily by theoretical assumptions, small proof-of-concept datasets, or highly constrained experimental conditions rather than robust multicenter prospective evidence. In some cases, these systems may demonstrate apparently strong technical performance under controlled settings while failing to maintain reliability across diverse clinical populations, imaging devices, or real-world healthcare environments (Ahmed et al., 2026).

Representative examples include fully autonomous treatment decision-making systems, LLM-only retinal diagnosis platforms, ungrounded GPT-based therapeutic recommendation systems, speculative digital twin retina simulations, and weakly validated multimodal causal inference frameworks. Although these approaches may appear technologically innovative, many currently lack the clinical robustness, explainability, and prospective validation necessary for safe deployment in ophthalmology (Singh et al., 2026). For example, autonomous therapeutic systems attempting to independently determine anti-VEGF treatment strategies without clinician oversight may introduce unacceptable safety risks because of limited contextual reasoning and insufficient handling of atypical clinical presentations.

Similarly, LLM-only retinal diagnosis or therapeutic recommendation systems remain highly vulnerable to hallucination, inaccurate clinical reasoning, fabricated references, and non-evidence-based outputs. While conversational AI and generative models may assist with workflow support or education, their use as primary diagnostic or therapeutic authorities in AMD remains inadequately validated. Digital twin retina simulations and multimodal causal inference systems are likewise biologically intriguing but remain largely theoretical because of insufficient longitudinal multimodal datasets, weak standardization, and limited mechanistic interpretability.

Several major risks are associated with Level V AI applications. These include hallucinated outputs, unsafe clinical recommendations, overfitting to small or biased datasets, lack of explainability, regulatory uncertainty, and poor reproducibility across healthcare systems. In addition, weak interpretability may substantially reduce clinician trust and complicate both ethical oversight and regulatory evaluation. Importantly, many speculative AI systems may inadvertently generate misleading confidence despite limited biological plausibility or inadequate validation.

At present, these approaches should be regarded primarily as exploratory research concepts rather than clinically actionable systems. Although some Level V technologies may eventually evolve into clinically useful tools with future methodological improvements, substantial translational barriers remain. Consequently, rigorous prospective validation, explainability enhancement, standardized benchmarking, regulatory oversight, and clinician-centered evaluation will be essential before such systems can be responsibly considered for clinical integration in AMD precision therapeutics.

The inclusion of Level V within the proposed framework is particularly important because it highlights the need for balanced and evidence-based interpretation of AI innovation in ophthalmology. As enthusiasm surrounding generative AI, multimodal learning, and autonomous medical systems continues to grow, distinguishing speculative technologies from clinically mature applications remains essential for ensuring safe, trustworthy, and scientifically responsible development of AI-enabled precision medicine.

## 7 Limitations and future research

### 7.1 Limitations

Despite the substantial promise of integrating AI with precision therapeutics in the management of AMD, multiple biological, technical, clinical, and ethical barriers continue to limit widespread clinical implementation. AMD is a highly heterogeneous and multifactorial disease influenced by complex interactions among genetic susceptibility, environmental exposures, systemic comorbidities, aging-related processes, and stochastic biological variation. Although genetic loci such as CFH, ARMS2/HTRA1, C3, and CFI have been strongly associated with AMD risk, their predictive utility in isolation remains limited because of incomplete penetrance and modest individual effect sizes. Furthermore, gene-environment interactions, including the influence of smoking, dietary factors, and metabolic conditions, remain incompletely understood, complicating efforts to establish robust biologically informed precision therapeutic strategies.

From a technical perspective, the performance and reliability of AI systems depend heavily on the availability of large-scale, high-quality, and well-annotated datasets. However, many currently available AMD datasets remain constrained by limited demographic diversity, imbalance in disease-stage representation, variability in imaging acquisition protocols, and inconsistent annotation quality. These limitations may introduce algorithmic bias and substantially reduce model generalizability across healthcare systems and patient populations. In addition, domain shift across imaging devices, institutions, and clinical environments remains a major challenge for real-world deployment. Differences in optical coherence tomography (OCT) platforms, image preprocessing methods, and grading standards may significantly affect model robustness and reproducibility. Moreover, the absence of standardized benchmarking frameworks and harmonized validation protocols complicates direct comparison between studies and limits reproducibility across the literature.

Although many AI models have demonstrated promising diagnostic and predictive performance, most systems remain based predominantly on retrospective single-center studies with limited prospective multicenter validation. Reported performance metrics are often difficult to interpret comparatively because studies vary substantially in imaging modalities, clinical endpoints, annotation methods, class balance, and validation strategies. In some cases, high algorithmic performance under retrospective experimental conditions may not translate into improved patient outcomes or clinically reliable real-world implementation. Challenges related to calibration instability, uncertainty quantification, explainability, and multimodal data fusion further complicate translational deployment. Consequently, prospective validation studies, standardized reporting frameworks, uncertainty-aware AI systems, and explainable AI approaches will be essential before widespread clinical integration can be safely achieved.

Clinical implementation also remains limited by workflow integration challenges and the incomplete maturity of precision therapeutic strategies. Genetic testing for AMD risk stratification or treatment selection has not yet become routine clinical practice because of cost considerations, reimbursement limitations, and the complexity of genomic interpretation. Similarly, AI-assisted decision-support systems require seamless interoperability with electronic health records, clinician training, and integration into existing ophthalmic workflows. Even when patients are successfully stratified, currently available therapies still demonstrate incomplete efficacy. A substantial proportion of patients exhibit suboptimal response to anti-VEGF therapy despite frequent injections, while complement inhibitors for geographic atrophy have shown only modest clinical benefit. In addition, stem cell therapies and gene-based interventions remain largely investigational, with unresolved concerns regarding long-term safety, scalability, durability, and regulatory approval. Thus, the current therapeutic landscape limits the immediate clinical impact of AI-driven precision stratification approaches.

Ethical and regulatory concerns further complicate the deployment of AI-enabled precision therapeutics in AMD. Increasing reliance on multimodal imaging, genomic information, and longitudinal patient data raises important questions regarding patient privacy, informed consent, cybersecurity, data governance, and equitable access to advanced technologies. Many AI systems continue to function as partially opaque “black-box” models, limiting interpretability and potentially reducing clinician and patient trust. Regulatory frameworks for AI-enabled medical systems are still evolving, particularly regarding accountability, liability, explainability, fairness, and post-deployment monitoring. Moreover, unequal access to advanced diagnostics, imaging infrastructure, and emerging therapeutics may widen disparities between high-resource and low-resource healthcare settings.

Overall, the translation of AI-enabled precision therapeutics into routine AMD care remains constrained by interconnected biological, technical, clinical, ethical, and regulatory challenges. Addressing these limitations will require interdisciplinary collaboration among ophthalmologists, data scientists, healthcare systems, regulatory agencies, and industry stakeholders. Future progress will depend on standardized multimodal datasets, robust prospective multicenter validation, explainable and uncertainty-aware AI systems, equitable implementation strategies, and clinician-centered deployment frameworks capable of supporting safe, reliable, and personalized ophthalmic care.

### 7.2 Future research

The integration of AI and precision therapeutics has the potential to fundamentally reshape the future clinical management of AMD. Future research is expected to increasingly emphasize clinically integrated, multimodal, and individualized approaches capable of supporting predictive, preventive, and personalized ophthalmic care. Within the proposed Five-Level Clinical Readiness and Translational Utility Framework, future developments are likely to evolve differently across levels, ranging from refinement of mature imaging-based systems to the emergence of more biologically informed precision therapeutic platforms.

At the Level I and Level II stages, future progress will likely focus on improving the robustness, interoperability, and real-world clinical integration of imaging-based and clinical decision-support AI systems. Prospective multicenter validation, standardized benchmarking protocols, uncertainty-aware prediction models, and explainable AI frameworks will be essential to improve generalizability and clinician trust. In addition, integration of AI-assisted therapeutic monitoring into electronic health records and ophthalmic workflows may facilitate more efficient longitudinal disease management, anti-VEGF treatment optimization, and personalized follow-up scheduling. Human-AI collaboration frameworks emphasizing clinician oversight rather than autonomous decision-making will likely become increasingly important for safe real-world implementation.

At the Level III stage, future research will likely emphasize multimodal precision therapeutic AI integrating genomic, transcriptomic, proteomic, metabolomic, molecular, and imaging information into unified analytical platforms. Such multi-omics approaches may provide deeper insight into the biological heterogeneity of AMD while enabling more refined patient stratification and individualized therapeutic prediction. The incorporation of polygenic risk scores and longitudinal multimodal biomarkers into AI systems may further improve early disease prediction, progression forecasting, and treatment-response modeling. As therapeutic options expand to include complement inhibitors, gene therapies, stem cell-based interventions, and sustained-release drug delivery systems, AI may play an increasingly important role in identifying optimal treatment candidates and supporting biomarker-guided therapeutic selection.

Emerging research directions may also include development of AI-enabled retinal “digital twins” and dynamic disease trajectory models capable of simulating individualized progression patterns and therapeutic responses. Although these approaches currently remain largely investigational and would presently fall within Level III to Level V categories, future advances in multimodal longitudinal data integration and computational modeling may eventually support more personalized *in silico* therapeutic planning. Similarly, foundation models, multimodal generative AI systems, and federated learning approaches may facilitate large-scale collaborative AI development while helping address data privacy and cross-institutional generalizability challenges.

At the Level IV stage, supportive and workflow-oriented AI systems are likely to expand within teleophthalmology, remote patient monitoring, automated documentation, and patient engagement platforms. AI-enabled home-monitoring devices, smartphone-based retinal assessment systems, and teleophthalmology infrastructures may support continuous disease surveillance and facilitate earlier detection of progression outside traditional clinical environments. These technologies may contribute to more proactive healthcare models by enabling individualized monitoring schedules, reducing unnecessary clinic visits, and improving accessibility for aging populations and underserved regions.

Importantly, future progress across all five translational levels must be accompanied by robust ethical, regulatory, and governance frameworks. Continued emphasis on explainability, fairness, transparency, patient privacy, cybersecurity, accountability, and equitable implementation will be essential to ensure safe and trustworthy deployment of AI-enabled precision therapeutics. Standardized reporting guidelines, prospective validation standards, and international collaborative frameworks may further support harmonized clinical evaluation and regulatory oversight across diverse healthcare systems and patient populations.

Finally, Level V AI applications currently characterized by insufficient evidence, poor interpretability, or speculative clinical utility may gradually evolve toward greater translational maturity as methodological rigor, multimodal integration, and prospective validation improve. However, cautious interpretation and evidence-based evaluation will remain essential to prevent premature clinical deployment of inadequately validated systems.

Overall, the coming decade is likely to witness increasing convergence between AI, multi-omics technologies, multimodal imaging, and emerging therapeutics, driving AMD care toward a more individualized, predictive, and proactive model of precision ophthalmology. By addressing current translational barriers and ensuring ethical, clinically grounded, and equitable implementation, AI-enabled precision therapeutics may not only improve visual outcomes in AMD but also serve as a broader prototype for future personalized medicine in ophthalmology.

## 8 Conclusion

AMD remains one of the leading causes of irreversible vision loss worldwide, with its multifactorial pathophysiology, substantial biological heterogeneity, and highly variable therapeutic responses continuing to pose major challenges for both clinical management and translational research. Recent advances in AI and precision therapeutics are increasingly reshaping the landscape of AMD care by enabling earlier disease detection, improved risk stratification, more accurate prognostic assessment, and increasingly individualized therapeutic strategies. Through the integration of multimodal data, including retinal imaging, genomic susceptibility profiles, molecular biomarkers, and longitudinal clinical information, AI systems may identify complex disease patterns and predictive signatures that are difficult to detect using conventional analytical approaches alone.

As illustrated in Figure 2, multimodal ophthalmic, molecular, and clinical data can be integrated within AI-driven analytical frameworks to support predictive modeling, therapeutic stratification, adaptive monitoring, and longitudinal clinical decision support. Such systems may facilitate both clinician-centered decision-making and patient-centered disease management within emerging learning healthcare environments. In parallel, precision therapeutic approaches leverage these multimodal insights to optimize treatment selection, monitoring strategies, and longitudinal disease management according to each patient's unique phenotypic and molecular characteristics, thereby supporting a gradual transition from reactive treatment paradigms toward more predictive, proactive, and personalized AMD care.

Despite these promising developments, most AI-enabled precision therapeutic systems for AMD remain at relatively early translational stages. Significant challenges continue to limit widespread clinical deployment, including limited external validation, predominantly retrospective study designs, dataset heterogeneity, domain shift across imaging platforms and institutions, explainability limitations, and insufficient prospective multicenter clinical evidence. Importantly, improvements in algorithmic performance metrics do not necessarily translate into improved patient outcomes or real-world clinical utility. Addressing these barriers will require standardized multimodal datasets, robust prospective validation frameworks, interpretable and trustworthy AI systems, careful regulatory oversight, and seamless integration into real-world ophthalmic workflows.

Looking forward, the convergence of AI, multimodal biomarker analysis, genomics, and next-generation therapeutics—including complement-targeted therapies, gene therapy, regenerative medicine, and stem cell-associated interventions—has the potential to fundamentally transform AMD management. Future AI systems may support dynamic therapeutic optimization, individualized treatment planning, longitudinal disease trajectory forecasting, and adaptive monitoring within continuously evolving learning healthcare systems. Although substantial translational and regulatory challenges remain, the integration of AI-enabled precision therapeutics into ophthalmology may ultimately facilitate safer, more effective, and more personalized AMD management while also establishing a broader framework for the future development of precision medicine across ophthalmic diseases.
